# Physiological predictors of reproductive performance in the European Starling (*Sturnus vulgaris*)

**DOI:** 10.1186/s12983-018-0288-3

**Published:** 2018-11-22

**Authors:** Melinda A. Fowler, Mélissa Paquet, Véronique Legault, Alan A. Cohen, Tony D. Williams

**Affiliations:** 1 Department of Biological Sciences, Simon Fraser University, 8888 University Dr, Burnaby, BC V5A 1S6 Canada; 2 Present address: Springfield College Biology, 263 Alden Street, Springfield, MA 01109-3797 USA; 3 Groupe de recherche PRIMUS, Department of Family Medicine, University of Sherbrooke, 3001 12e Ave N, Sherbrooke, QC J1H 5N4 Canada

**Keywords:** Dysregulation, European starling, Physiological complexity, Physiological state, Principal components analysis, Reproductive fitness, Statistical distance, *Sturnus vulgaris*

## Abstract

**Background:**

It is widely assumed that variation in fitness components has a physiological basis that might underlie selection on trade-offs, but the mechanisms driving decreased survival and future fecundity remain elusive. Here, we assessed whether physiological variables are related to workload ability or immediate fitness consequences and if they mediate future survival or reproductive success. We used data on 13 physiological variables measured in 93 female European starlings (*Sturnus vulgaris*) at two breeding stages (incubation, chick-rearing), for first-and second-broods over two years (152 observations).

**Results:**

There was little co-variation among the physiological variables, either in incubating or chick-rearing birds, but some systematic physiological differences between the two stages. Chick-rearing birds had lower hematocrit and plasma creatine kinase but higher hemoglobin, triglyceride and uric acid levels. Only plasma corticosterone was repeatable between incubation and chick-rearing. We assessed relationships between incubation or chick-rearing physiology and measures of workload, current productivity, future fecundity or survival in a univariate manner, and found very few significant relationships. Thus, we next explored the utility of multivariate analysis (principal components analysis, Mahalanobis distance) to account for potentially complex physiological integration, but still found no clear associations.

**Conclusions:**

This implies either that a) birds maintained physiological variables within a homeostatic range that did not affect their performance, b) there are relatively few links between physiology and performance, or, more likely, c) that the complexity of these relationships exceeds our ability to measure it. Variability in ecological context may complicate the relationship between physiology and behavior. We thus urge caution regarding the over-interpretation of isolated significant findings, based on single traits in single years, in the literature.

**Electronic supplementary material:**

The online version of this article (10.1186/s12983-018-0288-3) contains supplementary material, which is available to authorized users.

## Background

Reproduction, and specifically parental care, is widely assumed to be costly: expenditure of parental resources (time, energy, physiological capacity) on care of offspring increases offspring fitness at the cost to the parent’s ability to invest in self-maintenance, i.e. there is a fitness cost of reproduction [1, 2]. This trade-off between current reproduction and residual fitness (future fecundity and survival) is central to life-history theory [1, 3] even though empirical data to support this concept are surprisingly limited [4, 5]. It has long been assumed that costs of reproduction are mediated by physiological mechanisms associated with parental investment (e.g. [6–8]). However, we still know very little about the specific mechanistic basis of costs of reproduction, especially those associated with parental care [5, 9–11]. Furthermore, parental workload (offspring provisioning) shows large individual variation [12, 13], but the question of what mechanism(s) determines individual variation in workload during reproduction remains very poorly resolved [5, 14].

Much of the field of physiological ecology is based on the premise that fitness, fitness components, and key life history traits have coherent physiological underpinnings, and that we can thus understand how selection could act on life histories by understanding the mediating role of physiology [15, 16]. Indeed, there are a number of clear examples of exactly this kind of relationship, particularly with regard to hormonal control of reproduction, reproductive behaviour, and risk-taking (e.g. [17, 18]). However, the same clarity has not been evident in studies looking for physiological markers of individual quality or condition, and results on avian physiological costs of reproduction are equivocal. Although the potential physiological consequences of costs of egg production [19] and chick-rearing [7, 20, 21] have been documented, many studies focusing on energy expenditure have failed to reveal clear relationships with variation in parental care [22]. Some correlational studies have shown that variation in single physiological measures can be systematically related to workload or aerobic capacity [23–25], but see [26]. However, results from experimental studies with more comprehensive assessment of the physiological basis of costs associated with parental care in birds have been very mixed (reviewed in 5, e.g. [24, 27–31]). In general, these studies reveal few, or perhaps complex, relationships between workload (e.g. nestling feeding frequency) and physiology.

These conflicting results could be due to several factors. One potential issue is the lability of time point measurements and their relationship to environmental variability (see Discussion). Another factor may be the fact that physiological costs of reproduction may not be manifest in a single, current breeding attempt but might be deferred to later breeding or life-history stages [29, 32]. Additionally, there may be little covariation among physiological systems: e.g. immune function [33] or antioxidant levels [34]. Birds may be able to adjust individual components of their physiology independently [35–37] or there may be complex, context-dependent relationships among components [38, 39]. More broadly, this discrepancy suggests that ecologists have been somewhat naïve, simplifying complex, non-linear physiological systems into concepts (e.g. “oxidative stress,” “immunocompetence”) that may or may not have some basis in reality [38]. For example, despite knowledge of this complexity, research on oxidative stress in an ecological context usually uses one or two markers under the supposition that these can tell us most or all of what we need to know [40–42].

Data from multiple breeding attempts and a suite of physiological variables from multiple systems may shed light on these complex relationships. Moreover, a number of approaches have been proposed, mostly focusing on multivariate analysis of physiological markers to obtain a more stable, clear signal of underlying processes, principal components analysis (PCA) being the obvious first option, though it tends to identify, but not solve, the problem of multi-dimensional variation and instability of axes [33, 34, 43, 44]. Another noteworthy approach is a measure of body condition that uses Mahalanobis distance (DM), automatically adjusting for the correlation structure among biomarkers [45].

**Fig. 1:**
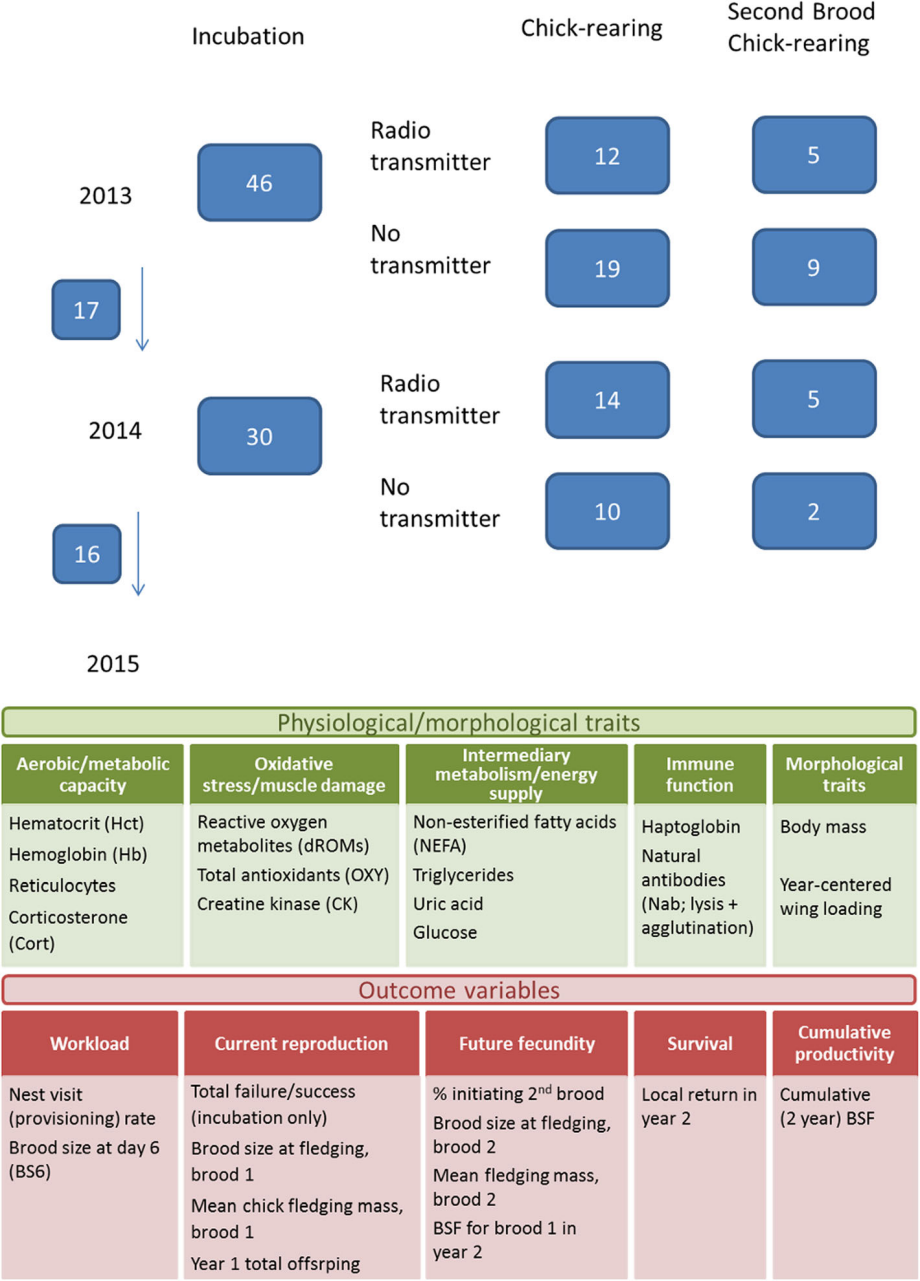
Experimental design. The figure presents sample sizes for each breeding stages at each year, and a summary of physiological and morphological predictor variables and life-history outcome variables measured. Numbers next to downward arrows indicate the proportion of birds that returned the following year

In this paper we analyze individual variation in physiological state in relation to parental care at two breeding stages (incubation and chick-rearing) in female European starlings (*Sturnus vulgaris*), for first-and second-broods in 2 years, using a repeated-measures design. European starlings are an ideal system in that they are highly synchronous breeders, minimizing variability due to time of year [14]. We assess the predictive capacity of 13 physiological variables at both stages reflecting four general physiological components: 1) aerobic/metabolic capacity, 2) oxidative stress and muscle damage, 3) intermediary metabolism and energy supply, and 4) immune function (Fig. 1). The physiological variables assessed here are common to many avian ecophysiology studies and we drew heavily from concepts embedded in the literature. A short description of our rationale follows. Hematocrit [35, 46, 47], hemoglobin [24, 48] and reticulocytes [49, 50] are common metrics for assessing aerobic capacity, and we included corticosterone (cort) in this category because baseline cort is an important metabolic hormone which has also been linked to breeding success [51–57]. Much recent work has linked measures of oxidative stress to life history evolution, fitness and cost of reproduction COR e.g. [25, 58–65], and we broadened this concept of “damage” by including creatine kinase, an indicator of muscle damage associated with flight [66, 67] Intermediary metabolites (nonesterified fatty acids (NEFA), triglycerides, glucose, and uric acid) are used in a great many studies to assess condition and feeding status in avian systems [68–78], e.g. birds with higher triglycerides are typically interpreted as being in positive energy balance [76], while elevated NEFA is thought to indicate a more catabolic state [75, 79]. Increased uric acid during foraging indicates the break down of dietary protein [72]. Uric acid is a by-product of protein catabolism, but can also function as an anti-oxidant [42]. Similalrly glucose is another metabolite that generally reflects a positive versus negative energy balance and whether a bird is regulating their carbohydrate metabolism appropriately [72, 75, 80]. Lastly, research into costs associated with immunocompetence is a very active field of research [29, 35, 37, 81], thus we chose several common metrics (haptoglobin and constitutive immune function, i.e. natural antibodies) to assess immune function.

Our overall goals were to assess whether the physiological variables are related to workload or immediate fitness consequences, and if they mediate future survival or reproductive success. Although workload variables (categorized here as nest visit rate and brood size; Fig 1) should reflect how hard females are working during parental care, the links may not be via energetics [22, 82, 83] and the consequences may be through other physiological avenues. Additionally, fitness costs indicate potential life history consequences: current reproduction, future fecundity (in second brood or in second year), survival and cumulative productivity (over 2 years; see Fig. 1).

Within this broad framework, we had several specific aims: 1) First, we consider correlations among the suite of physiological variables in both incubating and chick-rearing females, changes in these variables between stages, and repeatability. 2) We then test whether physiological state during chick-rearing is predictive of the workload for the current brood or is related to current or future fitness metrics. We incorporate not only the absolute physiological values, but also the change (delta) in physiological variables from incubation to chick-rearing, potentially reflecting physiological adjustments individuals make. 3) Finally, we generate integrated measures of physiological condition using multivariate approaches, under the hypothesis that the relevant signal may not be reflected in the levels of a single biomarker so much as in their joint distribution. We also perform multivariate analysis on the reproduction variables in an attempt to extract a stronger signal, and ask whether our negative findings with the individual markers could be attributable to a lack of appropriate multivariate analyses integrating them.

## Methods

Fieldwork was conducted at Davistead Farm, Langley, British Columbia, Canada (49°10′N, 122°50′W) between April-early July 2013–2014 and females were followed in 2014 and 2015 to measure maternal survival (local return rate) and fecundity in the year after blood sampling. This site comprises about 150 nest boxes mounted on posts around pastures and on farm buildings. Nest boxes were checked daily from April 1 to determine laying date, clutch size, brood size at hatching, at day 6 post-hatching, and at fledging (day 21), and mass and tarsus of all chicks was recorded when chicks were 17 days old. We then obtained the same data for second broods.

All blood samples were collected by puncturing the brachial vein with a 26½-gauge needle and collecting blood (< 700 μL) into heparinized capillary tubes. At the same time fresh blood was collected for a) hematocrit and hemoglobin measurements, b) two blood smears were prepared for reticulocyte counts, and c) glucose levels (mmol.L^− 1^) were measured with a handheld glucose meter (Accu-chek Aviva®; see Additional file 1). Blood samples were stored at 4 °C for up to 4 h before being centrifuged for 6 min at 10,000 *g*, plasma was collected and then stored at − 80 °C until assayed.

Although females were captured using different methods in incubation and chick-rearing (see below) all birds were blood sampled with 3 min of being handled. Incubation (day 6–8) samples from all breeding females were obtained by plugging the nest box hole prior to dawn. This method can result in different time periods that the birds are passively sitting in the nest box (mean 56 min, maximum 132 min) before being removed and blood sampled. We looked at possible effects of from when box was plugged early in the morning to when bird was removed on physiological variables and found no evidence for such effects (see Additional file 1: Figures S1 and S2). Incubating females were fitted with color bands and individually numbered metal bands (Environment Canada permit # 10646; males were not captured or banded, and thus, identity of males is unknown). As many females as possible were recaptured during chick-rearing of first broods (day 10–12 post-hatching) and again during chick-rearing of second broods (day 10–12). For a sub-sample of females (2013*, n* = 12; 2014, *n* = 14) we attached radio transmitters (Holohil Limited, Inc., model BD-2, mass = 1.9 g). There was no difference in mean brood size at fledging, mean 17-day chick mass, mean provisioning rate, or any physiological variable for females with and without radio-transmitters (*p* > 0.05 in all cases) so we pooled data for all subsequent analyses. During chick-rearing, females were caught using nest traps (Van Ert Enterprises, Leon, IA) as they entered nest boxes to feed chicks, removed immediately and blood sampled within 3 min.

We analysed plasma samples for 13 physiological variables using standard assay methods reported in previous studies; see Fig. 1 and Additional file 1 for full details. Due to variation in the amount of plasma we obtained, for some individuals there was not enough plasma to run all assays, thus sample sizes differ for some physiological variables. Raw data values (mean ± S.D.) for all physiological variables by year and breeding stage are given in Additional file 1: Table S1.

### Wing area measurements and wing loading

Wing surface area (chick rearing only) was calculated from digital photos taken in the field using the free image software IMAGEJ (available from http://rsbweb.nih.gov/ij/). We present chick-rearing wingloading only, as we were interested in the potential flight effects of provisioning, but were less interested in the flight ability of birds incubating eggs. Two wing photos were taken of the left wing spread on a board with 2 cm grid drawn. Each picture was scaled to the 2 cm grid using the software. The outline of the wing was traced in IMAGEJ two times for each photo, resulting in four measurements per individual. The coefficient of variation between measures in one photo was 0.39% and between two photos was 3.13%. Wing surface areas were averaged and doubled to attain total wing surface area. The body box (i.e. the area between the wings) was not included in the calculation of the wing surface area. Wing loading was calculated as mass/area [84]. Due to a difference in measurers by year, the control, chick-rearing values are mean centered within year.

### Measures of workload and fitness costs

We used two measures of workload, reflecting how hard the female must work (Fig. 1): nest visit rate (or provisioning rate) and day 6 brood size (i.e. brood demand during the linear phase of chick growth [85]). Nest visit rate was obtained from 30-min behavioral observations surveys between 09.00–14.00 on days 6, 7, and 8 post-hatching with 2–3 observations per nest (i.e. either 1-h or 1.5-h of data per nest [12]). We recorded number of visits for males and females separately, based on presence of bands/colour bands (we did not capture or band males). Visits where sex could not be determined were initially categorized as unknown, but unknown visits were quite low (~ 5–15% of total observations). Unknown visits were then partitioned between male and females based on the ratio of known visits, after Fowler and Williams [12].

Fitness metrics included current and future reproductive success, as well as survival. Specifically, the variables we used included current reproduction as a) brood size at fledging (day 21 post-hatching) and b) mean chick fledging mass (measured at day 17), for the first brood. In addition, for incubating birds only, we compared physiological variables among birds with total breeding failure or breeding success (≥ 1 chick fledged), and brood size at day 6 post-hatching, to assess immediate consequences of variation in incubation physiology. As measures of future fecundity we used, a) probability of initiating a second clutch (0/1), b) brood size at fledging (day 21 post-hatching) and mean chick fledging mass, for the second brood, and c) brood size at fledging for the brood in year 2. We present the percent of individuals initiating 2nd broods, but model the data as a logistic regression. Finally, we compared physiological variables during chick-rearing in year 1 to local return rate in year 2, and to the cumulative number of chicks fledged over 2 years in all breeding attempts (see Fig. 1).

## Statistics

### Pre-treatment of physiological variables

All data were analyzed using R versions 3.0.0, 3.2.1, and 3.2.2 (R Core Team 2015). We tested for normality and normalized all non-normal variables using either natural log or square root transformation (see Additional file 1). After normalizing data we tested for, but did not detect, any statistical outliers for any physiological variables except Cort. In univariate analyses, we excluded seven females which had Cort > 80 ng/ml, which is within the range of “stress-induced” levels in this species [52]. However, for multivariate analyses, we included these outliers to increase our sample size, and performed sensitivity analyses to assure it did not affect the results (see Additional file 1). The agglutination and lysis scores were combined using principal component analysis after Matson et al. [86] and we used the first axis (hereafter NAb PC1) in univariate analyses. We also performed a PCA on the antioxidant level (OXY) and the reactive oxygen metabolites (dROMs) measures (oxidative stress; see Additional file 1 for details). In multivariate approaches described below, we kept the two first PCs for both the NAb and the OXY-dROMs PCAs, because the biological interpretation is clearer: PC1 is activation of the system (higher levels of both markers) and PC2 is balance of the system (relative levels; see Additional file 1: Figure S3).

For individuals where we had physiological variable values during incubation and chick-rearing (for the first brood) we calculated the change in variable value (delta or ∆) between stages. For analyses of correlations among multiple variables, in incubating and chick-rearing birds, we considered results both using raw *p* values (*p* < 0.05) for exploratory purposes, and using adjusted *p* values in R based on False Discovery Rate (FDR) using the Benjamini/Hochberg correction [87]. Repeatability estimates were generated with the rptR package [88] and individual included as random after [12]. Physiological changes between incubation and chick-rearing were assessed with linear mixed effects models, including band and year as random effects.

### Physiological multivariate approach 1: Mahalanobis distance

We took two main approaches to summarize overall variation in physiology: PCA and DM. DM calculation has been extensively described elsewhere [45, 89], but briefly, it assigns a score of 0 (optimal condition) if the individual has the mean levels of all variables; scores increase as distance from this centroid goes up in multivariate space (the individual has a more “abnormal” profile). We decided to standardize (minus the mean and divided by the standard deviation) physiological variables by year and breeding stage; however, sample sizes for the four year-stage subgroups were too small to produce robust estimates of means for standardization (Additional file 1: Figures S4 and S5). We thus used regression to identify beta-coefficients with which to adjust year and stage means. Also, because DM requires complete data, we had hoped to exclude the reticulocyte variable (which has many missing observations), but DM was slightly more sensitive to inclusion/exclusion of this variable compared to other; we thus present analyses with (*n* = 80) and without (*n* = 104) reticulocytes included, and this sometimes changes results.

We generated different versions of DM, first a simple version of DM based on the population mean, and then more sophisticated versions based on a priori knowledge of physiological variables (see Additional file 1 for details). DM is defined as a distance from a centroid (supposed to represent the ideal physiological/homeostatic state), normally the population mean. Previous studies have found it to be a reasonable approximation, with little sensitivity to the precise definition [89, 90]. However, in this case we had a number of variables for which there was a clear a priori expectation that the optimal value was not the mean, in contrast to all previous studies. For example, low scores of the two OXY-dROMs PCs represent low activation of oxidative stress pathways and balance toward antioxidants rather than free radicals, respectively, so lower scores should be better, with no lower bound. For some other variables, we had a hypothesis about direction but no certainty. We thus used a combination of a priori knowledge and data driven methods to generate multiple versions of DM (approximately 20) based on which variables were considered optimum at their mean, minimum, or maximum. However, DM version has relatively little impact on our overall conclusions (see Additional file 1 for details), and we thus chose to present several of the most distinct, for ease of presentation. Additional file 1: Table S2 summarizes the final set of DMs that we kept for further analyses, including the basic one with year- and stage-specific means as the centroid. Additional file 1: Figure S6 shows correlations between these different versions.

### Physiological multivariate approach 2: PCA

We conducted PCA on all variables (including the two first PC axes for NAb and OXY-dROMs) with two versions: (1) PCA on centered and reduced variables (directional), and (2) PCA on the log of the absolute value of each variable centered and reduced (deviational, i.e., looking for a structure in the deviance, related to the theory underlying DM; see Additional file 1: Figure S7). However, the first axes explained less than 15% of the variance in both cases and examination of loadings did not provide a clear biological interpretation. Perhaps the combination of physiological complexity and small sample size given the number of variables made results unstable, or perhaps most variables were closely maintained within their homeostatic range, such that variation was not particularly meaningful; we nonetheless tested associations between the first three axes of both PCA and the performance variables for exploratory purposes. We have previously found that even when PCA axes explain a small amount of the variance and a biological interpretation is not clear, patterns can nonetheless be highly stable across populations and predictive of health status [91].

We also performed three PCA based on a priori knowledge, i.e. grouping the variables by physiological function (see Additional file 1: Figure S8). We performed PCAs on three groups with three variables and included the first axis for regression analysis: intermediary metabolism and energy supply (“metabolism/energy”), aerobic/metabolic capacity (“aerobic capacity”), and oxidative stress and muscle damage (“oxidative stress”).

### PCA on performance measures

We also generated a composite variable that summarizes variation in reproductive success. Due to missing data and biological complexities of the data structure (e.g. many individuals did not have a 2nd breeding attempt), we performed PCA on only 4 variables reflecting current breeding productivity: first brood size at day 6, first brood size at fledging (day 21), total young fledged in the year, and total mass of fledglings produced over the year. We kept only the first axis in further analyses (see Additional file 1: Figure S9).

### Relationship of physiological variables to workload and fitness components

First, univariate analyses of physiological predictors of workload and fitness components were conducted using lme4 package [92], for absolute physiological variable values at both breeding stages and for change between stages (delta incubation-chick-rearing). All models included year and individual identity as random effects. Body mass was included as a covariate in analyses on separate breeding stages. *P*-values were calculated with the Kenward-Rogers correction [93]. When brood size was investigated as a response variable, we used generalized mixed effects models with Poisson error distributions, and report the z-statistic and associated *p*-value. Binomial response variables (success, 2nd brood initiation, or survival) were analyzed as logistic regression.

In a second phase, we tested for associations between each composite physiological variable and the raw and composite performance variables. We conducted separate analyses on incubation physiology in relation to performance variables, chick-rearing physiology in relation to performance variables, and mean physiology across incubation and chick-rearing in relation to performance variables. We predicted that high DM would be associated with low reproductive success. We ran linear mixed effects models using the lme4 package [92] with the performance variable as the dependent variable, the physiological measure as the independent variable, and individual as a random effect (see Additional file 1 for details). Associations between DM and subsequent survival, we performed using logistic regressions (glm function). We interpreted these results in the light of general patterns across the analyses, not the significance of individual tests. Multiple testing is thus not formally adjusted for, but our approach is conservative overall.

## Results

### Correlations among the suite of physiological variables in both incubating and chick-rearing females, change between stages and repeatability

Here, we restricted our analysis of physiological data to samples obtained during incubation and chick-rearing for first broods due to the relatively small number of second brood samples. In incubating females, 22 pairwise correlations were significant at α = 0.05 among 13 physiological variables (Additional file 1: Table S4), but only 6 after FDR adjustment: dROMs correlated positively with OXY and negatively with uric acid, and haptoglobin correlated negatively with reticulocytes, OXY, dROMs, and positively with CK (all *p* < 0.01). In chick-rearing females, all 13 physiological variables were independent of body mass and year-centered wing loading (Additional file 1: Table S5). Ten pairwise correlations were significant at α = 0.05, but only two relationships remained significant after FDR adjustment: hematocrit and hemoglobin (positively), as well as haptoglobin and dROMs (negatively).

**Fig. 2:**
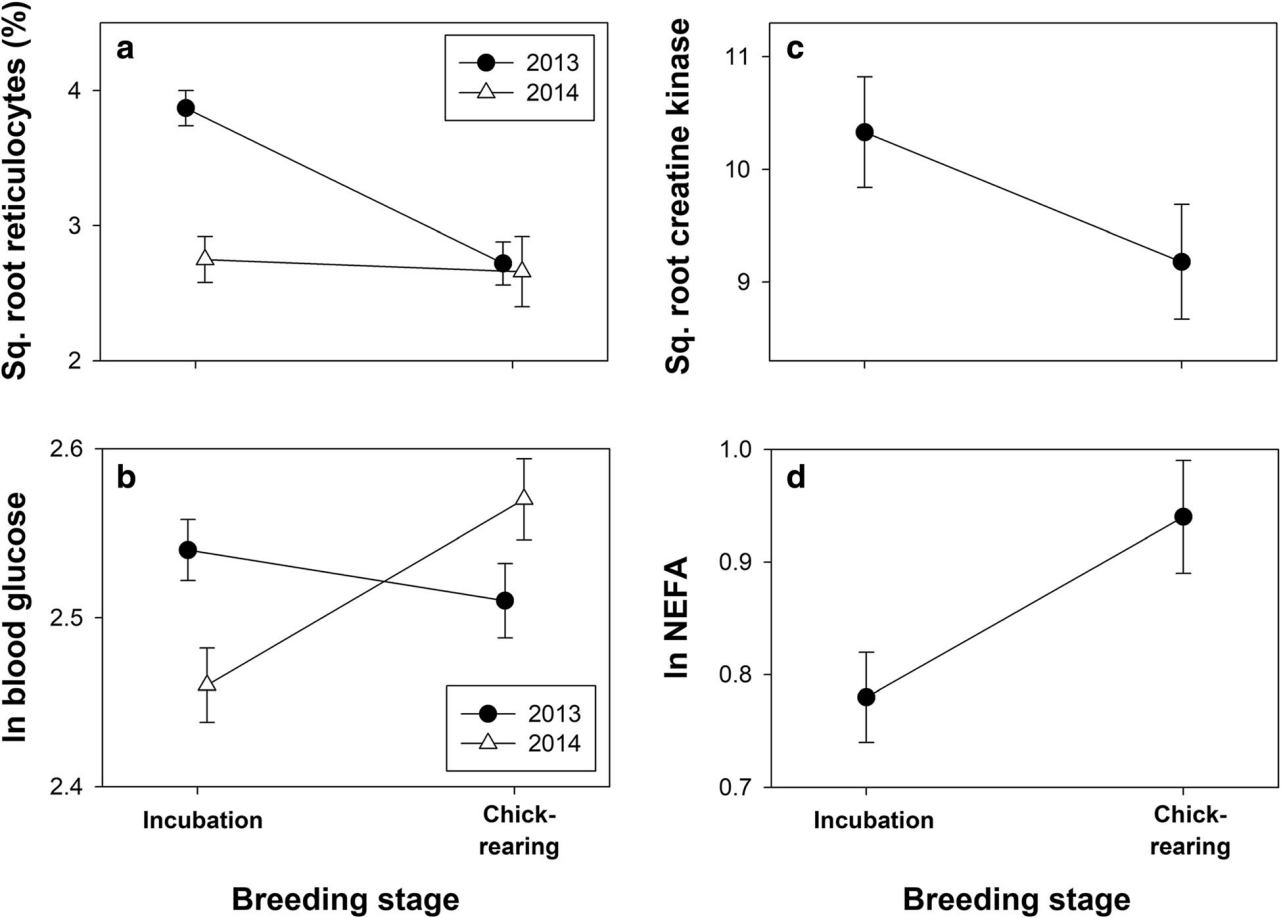
Representative examples of patterns of change in physiological traits showing breeding stage*year interactions: **a**) reticulocyte counts, and **b**) blood glucose levels, and traits showing an effect of breeding stage with no interaction: **c**) plasma creatine kinase and **d**) plasma non-esterified fatty acids; see text for details

For three physiological variables there was a year*breeding stage interaction (*p* < 0.001). Reticulocyte counts decreased from 15.6 ± 6.1% in incubating birds to 8.2 ± 5.1% during “chick-rearing” in 2013 but did not change between incubation (8.7 ± 5.9%) and “chick-rearing” (7.3 ± 2.5%) in 2014 (Fig. 2a). Secondly, blood glucose decreased between breeding stages in 2013 and increased between stages in 2014 (Fig. 2b). Finally, OXY increased from incubation to chick-rearing in both years but the change was much greater in 2014 (167.6 vs 232.9 μmol HClO.ml^− 1^; + 39.0% compared with 2013 (254.4 vs. 271.1 μmol HClO.ml^− 1^; + 6.6%).

**Fig. 3:**
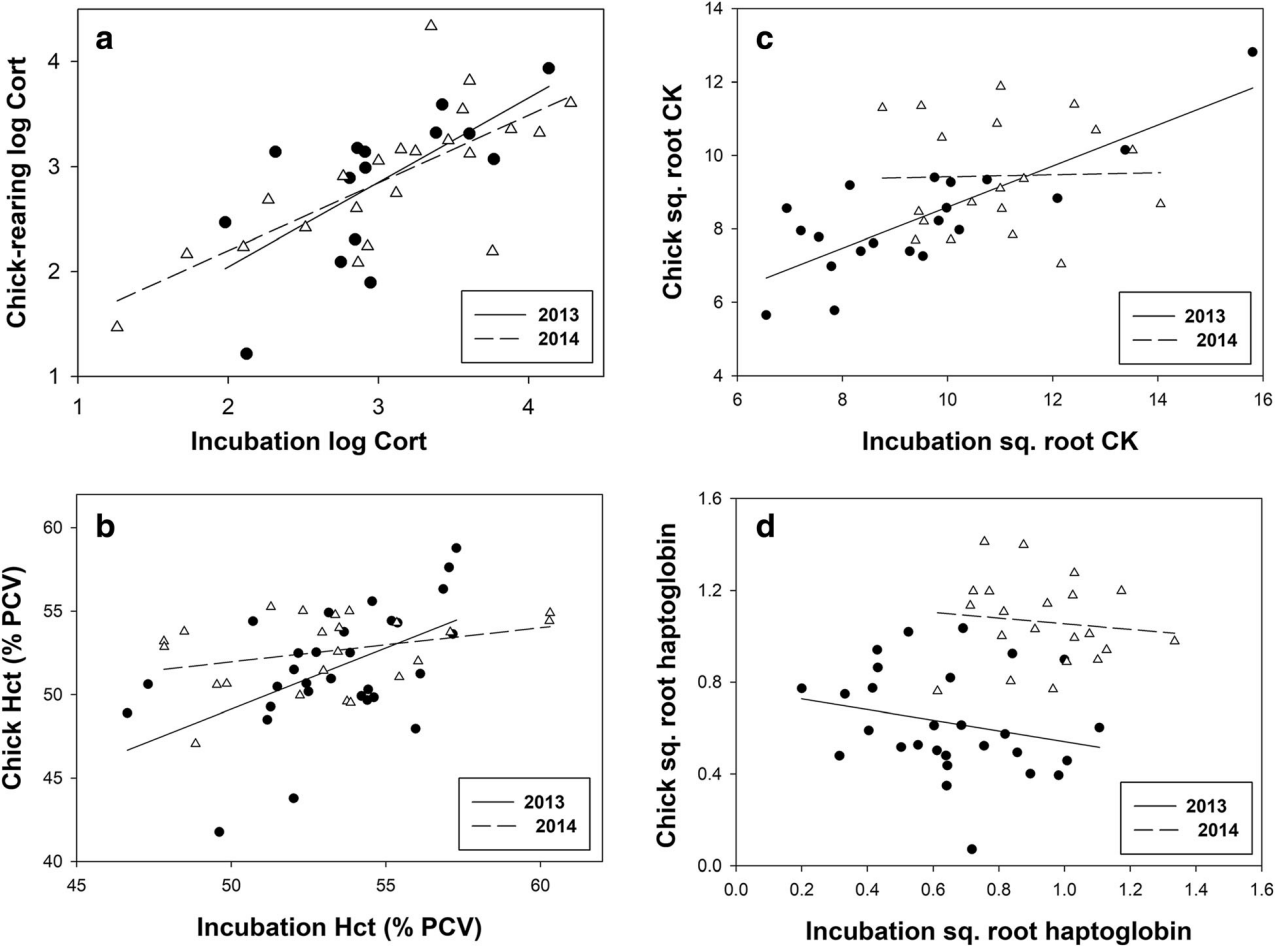
Representative examples of patterns of repeatability in physiological traits: **a**) plasma corticosterone, **b**) hematocrit, **c**) plasma creatine kinase, and **d**) haptoglobin; see text for details

For six variables there was a significant effect of year, but no year*breeding stage interaction: hemoglobin (*p* < 0.01), CK (*p* = 0.04), dROMs (*p* < 0.001), NEFA (*p* = 0.04), uric acid (*p* = 0.03) and haptoglobin (*p* < 0.001). We therefore included “year” as a random factor in analysis of breeding stage differences for the 10 variables with no year*stage interaction. Chick-rearing birds had lower body mass (*p* = 0.04), hematocrit (*p* = 0.03), dROMs (*p* = 0.03), and plasma CK (*p* < 0.01; Fig. 2c) but higher hemoglobin (*p* = 0.03), NEFA (Fig. 2d), triglyceride and uric acid (all *p* < 0.001). There was no difference in log Cort, haptoglobin or NAb PC1 among breeding stages or years. The only physiological variable that was repeatable between incubation and chick-rearing in *both* years was Cort (2013, *r* = 0.67, *p* = 0.03; 2014, *r* = 0.69, *p* < 0.001; overall *r* = 0.55; Fig. 3a). Hematocrit was repeatable in 2013 (*r* = 0.42, *p* = 0.01) but not in 2014 (*r* = 0.03, *p* = 0.09; Fig. 3b). Similarly, CK was highly repeatable in 2013 (*r* = 0.75, *p* < 0.001) but not in 2014 (*r* = 0, *p* > 0.70; Fig. 3c). For some variables year differences would have generated “apparent” repeatability if pooled data were analysed (e.g. Fig. 3d).

### Relationship between univariate physiological state and workload and fitness costs

We found that physiological state during incubation did not predict BS6 (*p* > 0.05 in all cases). Additionally, there was no difference in physiological variable values for incubating birds among individuals that subsequently showed total breeding failure versus breeding success (≥ 1 chick; logistic regression, *p* > 0.14 in all cases).

There were significant relationships for only 4/156 (2.6%) pair-wise contrasts between chick-rearing physiological variables and life-history outcomes, and only two if the three females with BSF = 0 were excluded from the analysis (see Table 1 for summary of results): Cort was positively associated with nest visit (provisioning) rate and NEFA negatively with mean fledging mass for the current brood (brood 1). There were no significant relationships between any physiological variables and any measure of future fecundity, survival and cumulative productivity (*p* ≥ 0.05 in all cases). Using change in physiological variable value, there were only 5/168 (3.0%) significant pair-wise contrasts with life-history measures, and, again, only two after excluding females with BSF = 0 (Table 1): change in mass and in triglyceride levels were negatively associated with provisioning rate and mean fledging mass of chicks in first broods, respectively.

**Table 1:** Summary of relationships between chick-rearing physiology, workload, current productivity, future fecundity and survival

Life-history stage	Outcome variable	Absolute physiological trait value (chick-rearing)	Change in physiological trait value (incubation-chick rearing)
Workload	Nest visit rate	Cort ↑ (*F*_1,9_ = 7841, *p* < 0.001, *n* = 43)	Mass ↓ (F_1,12.4_ = 6.5, *p* = 0.03, *n* = 51)
	Brood size at day 6	–	–
Current productivity	BSF1	dROMs ↓ (z = − 1.95, *p* = 0.047, *n* = 53)^a^	–
	Mean fledging mass, brood 1	NEFA ↓ (F_1,46.1_ = 6.6, *p* = 0.01, *n* = 50)	Trig ↓ (F _1,45.1_ = 5.9, *p* = 0.02, *n* = 48)
	Year 1 total offspring	dROMs ↓ (z = − 2.17, *p* = 0.03, *n* = 52)^a^	Trig ↑ (z = 2.1, *p* = 0.04, n = 52)^a^
Future fecundity	Probability initiating 2nd brood	–	–
	BSF2	–	OXY ↑ (z = 2.1, *p* = 0.04, *n* = 16)^a^
	Mean fledging mass, brood 2	–	–
	BSF1 in year 2	–	–
Survival	Local return rate, year 2	–	–
Cumulative productivity	No. fledglings over 2 years	–	Trig ↑ (z = 2.5, *p* = 0.01, *n* = 12)^a^

Direction of associations is indicated by arrows: up arrow for positive associations and down arrows for negative ones

^a^ Not significant (*p* > 0.05) when n = 3 females with BSF = 0 are excluded

### Relationship between multivariate physiological state and workload and fitness costs

**Fig. 4:**
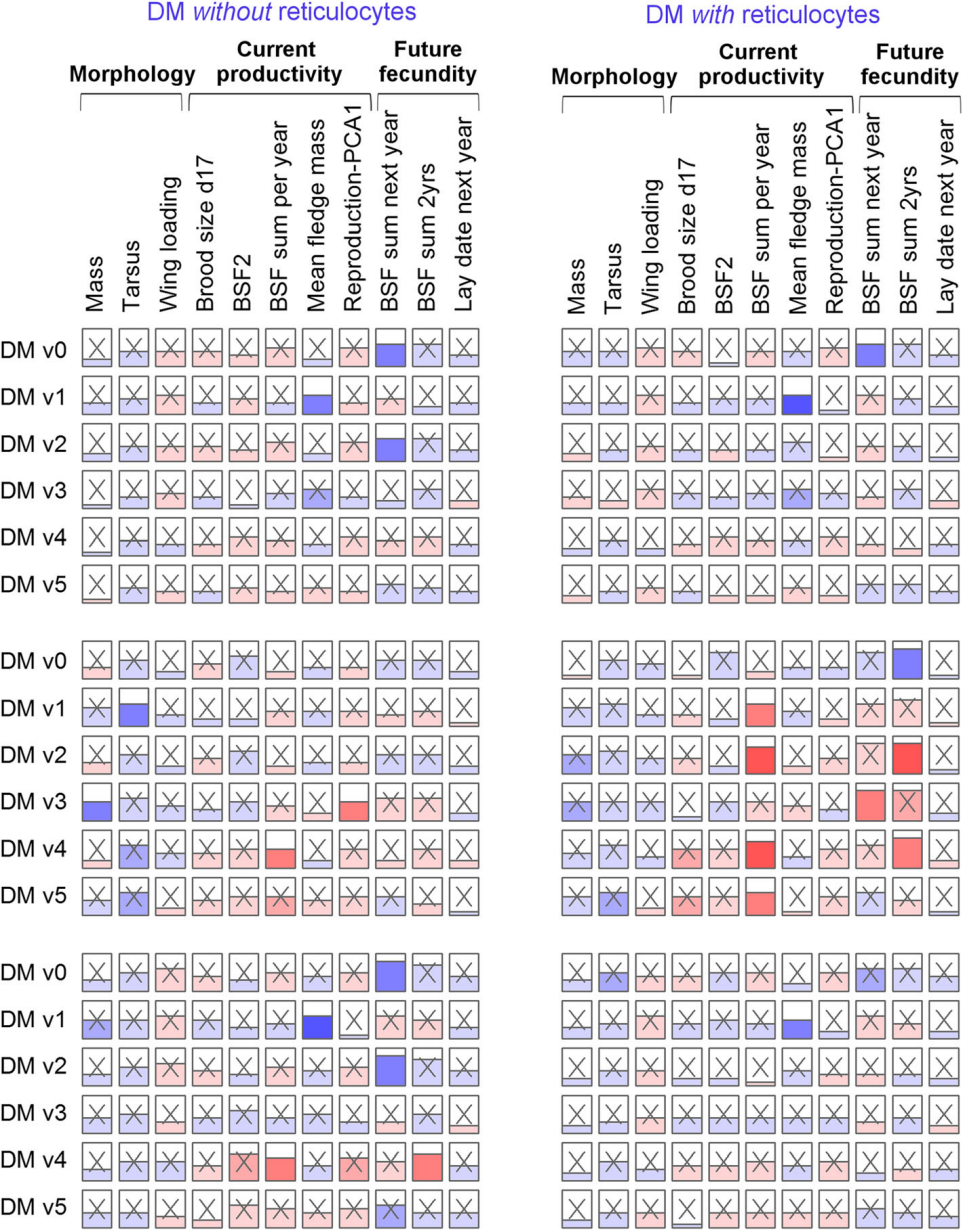
Few associations between physiological summary variables (DM) and reproductive performance. We performed regression models to evaluate the effect of different DM versions, excluding reticulocytes or not (*n* = 104 and 80 respectively), on each performance measure, using observations during the incubation stage (**upper panel**), the chick-rearing stage (**middle panel**), or the mean of all available observations within a year (**lower panel**). DM v0 was calculated using the mean as the centroid for each biomarker, DM v1-v4 represent versions based on a priori hypotheses, either using the mean + 3 standard deviations (high) or the mean – 3 standard deviations (low), and DM v5 was generated by random choice of mean, high (mean + 3 standard deviations), or low (mean - 3 standard deviations) for the centroid definition (see Additional file 1 for details). Height of the horizontal line in the box represents the effect size and the color underneath is blue for positive effects and red for negative ones, with color hue illustrative of *p*-values (darker shades for lowest *p*-values). Non-significant coefficients are marked with an “X”

Here, we pooled first and second brood chick-rearing data into a single chick-rearing category to increase our sample size and to ensure that we fully captured the breeding success of the most successful individuals. We ran analyses excluding second broods and obtained highly consistent results (data not shown). Overall, DM appears to be largely unassociated with performance variables (Fig. 4). Only 23 tests of 396 in Fig. 4 (5.8%) were significant at α = 0.05, not much more than expected by chance. Moreover, the tests are not independent (there are strong correlations among DM versions and among performance measure subsets, see Additional file 1: Figure S6). During chick-rearing, four of six DM versions show a clear negative association with total young fledged in a year (BSF sum per year) when the analysis includes reticulocytes, but this is replicated in only one DM version after excluding reticulocytes (Fig. 4), and is not replicated for incubation values. Further, this finding is not broadly confirmed by other reproduction variables, including the reproduction PC axis, fledgling mass, intermediate brood sizes, or subsequent year lay date. DM was not associated either with survival (Table 2).

**Table 2:** Survival analysis through logistic regression with local return next year, for six versions of DM (v0-v5)

DM (*n* = 35)	Survival			
	OR	LCI	UCI	*p*
v0	0.917	0.415	1.973	0.822
v1	1.126	0.532	2.445	0.753
v2	0.812	0.335	1.906	0.633
v3	0.744	0.299	1.763	0.505
v4	0.939	0.434	2.007	0.869
v5	0.816	0.382	1.644	0.572

DM v0 was calculated using the mean as the centroid for each biomarker, DM v1-v4 represent versions based on a priori hypotheses, either using the mean + 3 standard deviations (high) or the mean – 3 standard deviations (low), and DM v5 was generated by random choice of mean, high (mean + 3 standard deviations), or low (mean - 3 standard deviations) for the centroid definition (see Additional file 1 for details).

**Fig. 5:**
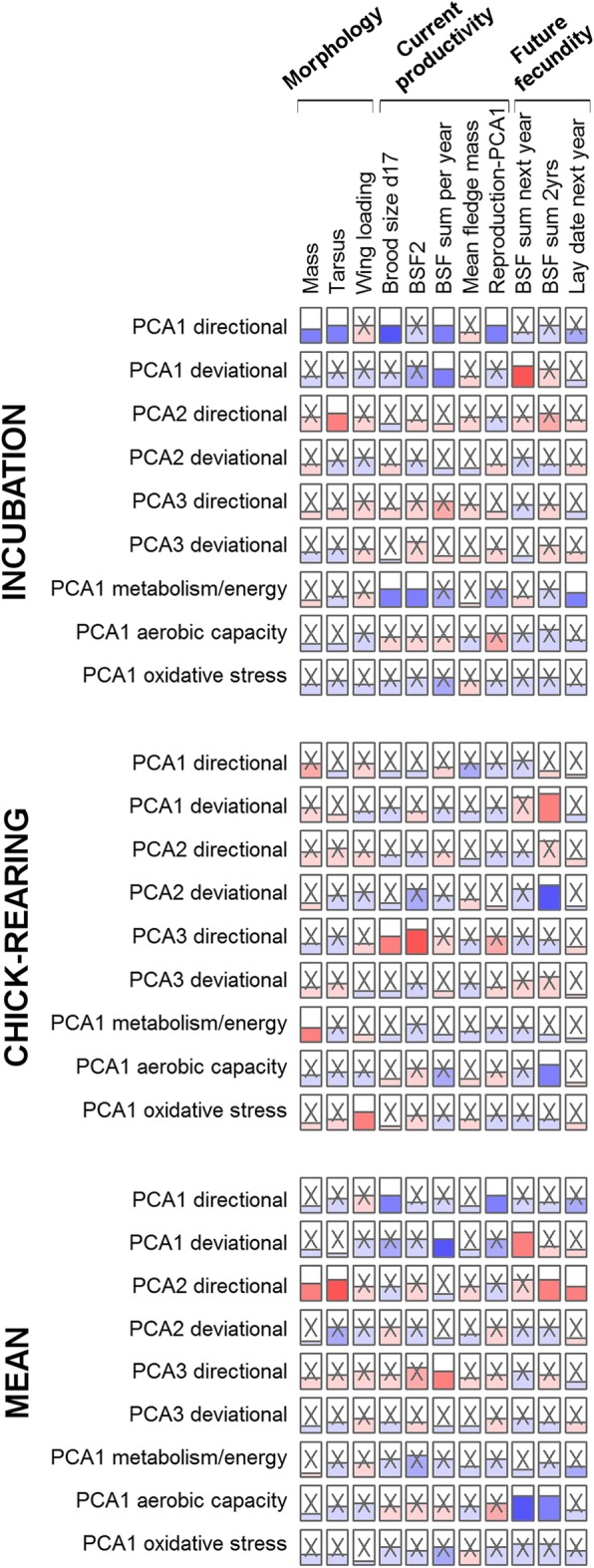
Few associations between physiological summary variables (PCA axes) and reproductive performance. We performed regression models to evaluate the effect of the first three axes of the PCAs on all centered and reduced physiological variables (PCA directional, *n* = 80), on the logarithm of their absolute values (PCA deviational, n = 80; see Additional file 1: Figure S7), and the first axis of PCAs on functional groups of physiological variables (*n* = 133, 92, and 122 respectively for metabolism/energy, aerobic capacity, and oxidative stress functional groups; see Additional file 1: Figure S8), on each performance measure, using observations during the incubation stage (**upper panel**), the chick-rearing stage (**middle panel**), or the mean of all available observations within a year (**lower panel**). Height of the horizontal line in the box represents the effect size and the color underneath is blue for positive effects and red for negative ones, with color hue illustrative of *p*-values (darker shades for lowest *p*-values). Non-significant coefficients are marked with an “X”

Similarly, analyses relating physiological PCs to performance variables (Fig. 5) showed no robust associations. Some associations were significant, but there were no clear patterns and most or all of these are likely false positives due to the large number of tests performed (29 of 297 (9.8%) significant at α = 0.05). Not a single positive result was replicated between incubation and chick-rearing analyses. Of course this does not exclude the possibility of some true positives among our significant results – PCA1 directional during incubation and PCA3 directional during “chick-rearing” seem to have disproportionate numbers of significant associations, for example – but the consistency of the findings is insufficient for us to draw firm conclusions.

## Discussion

Our overall goals were to assess whether physiological variables are associated with workload ability or immediate fitness consequences (current reproduction) and if they may potentially mediate future fitness components (future fecundity or survival). Below, we discuss three main results including 1) covariation among physiological metrics, 2) changes over stages and year and 3) lack of relationship between physiology, workload and fitness components.

We found little evidence for covariation among our suite of 13 physiological variables either in incubating or chick-rearing birds and even within functional groups of variables, suggesting that there is little stable covariation not only among physiological components of the same physiological system (e.g. immune function), but also among different physiological systems. The lack of stable covariation in variables does not necessarily indicate a lack of functional relationships, but rather relationships that are highly contingent on individual circumstances. Flexibility among physiological components may enable the individual to adjust to a changing environment [35, 38]. Thus, it is critical to consider both the physiological constraints as well as the ecological context when examining patterns of covariance among phenotypic traits.

There is also increasing evidence that there is little covariation among physiological traits, even for components of the same physiological system [33, 34]. Birds appear to adjust individual components of their physiology independently [35–37], and there can be complex, context-dependent relationships among components [38]. We did find one marker that was correlated with multiple others in incubating birds: haptoglobin was negatively correlated with reticulocytes, dROMs and OXY and positively correlated with CK. Haptoglobin is an acute phase protein that scavenges hemoglobin released into the circulation by hemolysis or normal red blood cell turnover [86, 94]. Incubating females show a particular type of anemia due to the challenges of egg-laying [46, 49, 95] and this, combined with regenerative erythropoiesis, involves increased red blood cell turnover. Therefore, our results suggest that the effects of reproductive anemia, perhaps mediated by haptoglobin, might extend to several other physiological systems in post-laying females.

Our exploration of changes between stages and year indicated that several physiological variables changed from incubation to chickrearing stage, but few changes were consistent between years and for some there was a stage by year interaction. We assessed repeatability between stage and year and found only baseline cort to be repeatable between stages in both year. Hematocrit and CK were repeatable in 2013 but not 2014. Interestingly, our data on variation with breeding stage differ from those of Kern et al. [27] who reported that in female pied flycatchers plasma triglyceride and glucose levels were not different between incubating and chick-rearing birds, but that incubating birds had higher uric acid and free-fatty acid levels. In contrast, we found that chick-rearing birds had higher plasma triglyceride and uric acid levels, likely due to chick-rearing birds actively feeding and metabolising food items with lipid and protein. Our results further highlight the fact that a “true” baseline for physiological values in free-ranging individuals may not exist. These individuals were likely experiencing annual variation or demonstrating individual differences in physiological flexibility in different ecological contexts.

Indeed, we also found annual variation in physiological variable values, even within breeding stages, lack of repeatability for most variables, and even breeding stage*year interactions. Repeatability in single samples taken between stages and years (as we do here) has been a tool for many avian eco-physiologists [54, 96–98]. While this approach has acknowledged shortcomings (discussed below), there are also examples of relationships among single samples and reproductive success [98, 99]. Suggested improvements on this sampling methodology include a reaction norm approach [97, 100], which we employ with our analysis of ‘delta’, or changes between stages, as a predictor variable and including information on the environmental context [98].

Our main finding, given the original goals of this study, is that we could predict very little of the variation in workload or fitness costs with physiological metrics, either as raw metrics or as the change in physiology between breeding stages, and even with composite physiological variables using several multivariate methodologies. It is true that our metric of workload (i.e. nest visit rate) may not capture the full picture of the work being done by the parent. We recommend an area for future research would include quantifying distance traveled to give a more fine scale look at workload [14]. Given the number of tests we have performed, the underwhelming consistency, and the underwhelming *p*-values, we cannot draw a clear conclusion for an association. Physiology as a level of organization has long been a key component of the organismal performance paradigm [101]. Life history trade-offs are complex and often mediated through multiple axes, such as immunocompetence, energetics and endocrinology [15]. Some linkages are more direct than others that may be a result of physiologically based internal resource allocation, while yet another possibility is that the fitness outcome is mediated through behavior [15, 102]. Careau and Garland [103] designate physiology and biochemistry as lower-level traits and suggest that it is unlikely that these directly influence Darwinian fitness without intermediate effects on performance, behavior, and/or energetics. One hypothesis is that natural selection generally acts most directly on behavior, less on performance abilities, and least directly on lower-level morphological, physiological, and biochemical traits [101, 103, 104]. The concept that individual variation in physiological traits is filtered through behavior and performance may explain why we were unable to detect direct relationships between physiological variables and life history variables.

There are some clear limitations to our study. The environmental context the birds are experiencing has the potential to contribute to the lability in physiological values. The birds in this study are in two distinct metabolic states: incubation (where they had been fasting overnight) and chick-rearing where they are feeding continuously. Because the chick-rearing birds are in a continuously feeding state, there is little concern for a single food item causing a large perturbation to metabolites. Instead, the levels measured should provide a more integrative view of their metabolism. Metabolites in feeding birds are a very common metric to indicate fattening/fuel status [76]. Several studies have used experimental methodology controlling fat content and daily food intake and confirm that triglyceride is a robust metric to indicate mass gain and fuel status [69, 105–107]. While some may fluctuate more than others, the fact that some (i.e. glucose) remain stable indicates appropriate homeostatic mechanisms are functioning [108].

Environmental variation could certainly be responsible for the instability of our results. The two study years did differ markedly in terms of ecological context. In 2013 mean laying date was relatively early and overall breeding productivity was below-average (a “poor” year), and in 2014 mean laying date was relatively late and breeding productivity was above average (a “good” year) compared with our long-term (15 year means). A preliminary analysis showed very few correlations with temperature or rainfall during the days prior to physiological sampling or annual differences and with only 2 years of physiological data we are unable to speculate further.

Another limitation is our inability to measure how much the physiological variables fluctuate short-term (within-breeding stage repeatability). Single point samples of physiological variables such as hormones have the potential to vary based on environmental context (e.g. predators, weather, conspecifics-see [109] and indeed they are crucial to an individual’s ability to be able to respond appropriately to the current conditions. This may lead to fluctuating values and limited repeatability estimates [110]. Flexible traits that enable individuals to respond to the environment may evolve through natural selection without a significant repeatability estimate [111]. The single sample approach may bring variability that is difficult to interpret but, relationships among single sample values and fitness components have been found [54, 98, 99]. The incorporation of a reaction norm approach to aid in interpretation has been made by multiple authors [97, 100, 109, 112].

The canalisation hypothesis suggests that variables that are more linked to survival are regulated within a narrow, ‘optimal’ window, while others that are less related to survival are allowed to fluctuate [39]. This is also consistent with work on metabolic systems [108]. Future analyses would be needed to explore this in detail, though we note that we found no clear association, regardless of the coefficients of variation of the individual physiological variables.

Despite these limitations, there are numerous examples of specific physiological adaptations associated with extreme performance (reviewed in [113]). Detecting these associations may depend on whether or not the individuals are motivated to perform maximally and the ecological context in which they must perform a relevant task [114, 115]. Despite the correlational nature of our study, we had a very large range of individual variation in the ‘task’ performed, i.e. rearing offspring: some individuals made only one breeding attempt, had no breeding productivity, and did not return the following year, whereas other individuals reared up to 10 chicks from two broods, returned the following year, and reared up to 8 more chicks from two further broods. Accompanying the variation in success, we observed a nine-fold variation in workload (nest visit rate). It is therefore surprising that we see no physiological signature of this marked individual variation in performance and reproductive success. This highlights a striking contradiction with the widely held views that costs of reproduction are presumably associated with higher levels of reproductive investment (i.e. larger clutch size), and are widely assumed to involve physiological costs [5]. Alternatively, if birds can adjust multiple individual components of their physiology independently [33–35], there might be many physiological paths to fitness. In fact, recent work indicates that if birds are experimentally challenged to work harder (weights and wing-clipping), a physiological cost of reproduction is detectable [30]. Thus, birds that operate in ‘normal’ circumstances may not be under the maximal motivation that elicits a physiological signal linked to a cost of reproduction.

It is of course possible that our negative results are due to a lack of sufficient data or appropriate approaches to detect real patterns, or to other limitations with physiological measurements (see above). If our sample size had been much larger, if we had used another technique for integrating variables (or just the right tweak on our methods), or if we had better biomarkers, we might well have detected a clear pattern. But if this is the case, it is not encouraging for our ability to detect such signals in future studies: as can be seen here, the more methods are tried, the harder it becomes to correctly identify a true positive among the false positives certain to arise through multiple testing. Even if our negative results are due to the lack of appropriate methods, they still have important biological implications: if there were a clear, simple, straightforward signal, we would have detected it. We relied heavily on previous research exploring the relationship between metabolites and fueling and reproductive investment, as well as oxidative stress, aerobic capacity and performance. Many of our biomarkers have shown associations with aspects of life histories or performance in other studies [24, 29, 46, 49, 50, 53, 54, 59, 63, 67, 69, 74, 81, 107, 116–119] (and see Introduction).While we acknowledge clear limits to the physiological theory necessary to appropriately select physiological variables in a study such as this, our chose of variables was informed by the literature to the extent possible.

We used a method of multi-variate analysis with DM as an ecophysiology measure, which did identify clear associations between DM and both metabolic capacity and a foot inflammation score in red knots (*Calidris canutus*), each in the expected direction [45]. However, those performance variables were more directly related to health status, not fitness. While it is certainly possible that, despite our previous finding with the red knots, DM is not capturing a signal relevant to anything important in the starlings, another intriguing possibility is that DM is accurately capturing health status or condition, but that condition has no straightforward association with the reproductive variables we measured here. One potential reason for the discrepancy between this study and our previous work on DM is the substantial expected and observed physiological variation within our population (but see our comments re. limitations above). While our total sample was relatively large, sub-samples by year and breeding stage were not, and many of our measures differed markedly across these sub-groups. In contrast, red knots were captive during that study and were measured ~ 12 times each, reducing variation. DM has also performed very well in human populations, with numerous replications across datasets and biomarker combinations (e.g. [90, 120]. The principle of DM relative to homeostatic control requires a relatively homogeneous population, and this was likely not sufficient in the present study, in contrast to humans and red knots. Our best guess is thus that DM might still perform well for physiologically homogeneous populations (same breeding stage, parasite pressure, etc.) but may break down as variation in optimal physiological state increases, perhaps related to more highly variable ecological context (as well as life history context). For example, DM might have performed better had we looked only at a single year and breeding stage, with a sufficient sample size to properly calibrate the technique. Despite being a highly synchronous population in a relatively small area, as we have seen from our data, this is not necessarily a homogeneous population.

We are aware that some readers may find some of our analyses either intimidating or undirected and too exploratory. If we had claimed to have a clear positive result, it would indeed be critical to consider the possibility of a false positive in relation to multiple testing. However, the univariate analysis demonstrated that although we based our choice of physiological variables on previous studies, current knowledge about the relationship between physiology and fitness is insufficient to provide targeted testable hypotheses, necessitating some level of exploratory analysis. For example, in defining different versions of DM, we could not always agree among co-authors whether the optimal value for a given marker was high, low, or intermediate. In this sense, the core take-home message is that even with such an exploratory approach and so much effort put into the statistical analyses, there is no clear biological signal. This is not an encouraging message for ecophysiology, but it is not one that can be ignored simply because it is inconvenient. Going forward, it will be important to try to replicate our negative finding using other integration approaches, species, and biomarkers. There may be particular factors related to our study that caused a negative result for an approach that might be promising more broadly. The positive result in red knots was replicable across many alternative model specifications, and therefore appears to be robust within that dataset.

## Conclusions

We feel this study provides a strong warning to eco-physiologists hoping to use physiological measures to quantify body condition or individual quality, particularly in cases where there may be substantial physiological or environmental heterogeneity in the population. Observational studies, while best representing the experience of the free ranging animal, may be especially susceptible to this, while experimental controls may lessen this heterogeneity. On the other hand, observational studies have the advantage of studying only natural conditions, an important point given the interconnectedness of the underlying physiological mechanisms, and therefore the difficulty of making inferences piecemeal.

## Additional file

Additional file 1: Physiological predictors of reproductive performance in the European Starling (*Sturnus vulgaris*). (DOCX 2220 kb)

## Abbreviations

BSF: Brood size at fledging
BSF1: BSF for first brood only.
BSF2: BSF for second brood only
CK: Creatine kinase concentrations, (U/L)
Cort: Corticosterone (ng/mL)
DM: A measure of body condition based on statistical distance (Mahalanobis distance)
dROMs: Reactive oxygen metabolites (mg H2O2/dL)
NAb PC1: First PCA axis on AG and lysis variables, reflecting the immune function
NAb: Natural antibody titers
NEFA: Non-esterified fatty acids (mmol/L)
OXY: Antioxidants titers (μmol HClO/mL)
PCA: Principal components analysis
