# Supplementary material for: Physiological predictors of reproductive performance in the European Starling (Sturnus vulgaris)
Source: Front Zool. 2018 Nov 22;15:45. doi: 10.1186/s12983-018-0288-3 (PMC6249724; doi:10.1186/s12983-018-0288-3)
Supplement: Supplementary file 1 — Physiological predictors of reproductive performance in the European Starling (Sturnus vulgaris). (DOCX 2220 kb) [file 12983_2018_288_MOESM1_ESM.docx]

***Additional file 1***

**Physiological predictors of reproductive performance in the European Starling (*Sturnus vulgaris*)**

Melinda A. Fowler, Mélissa Paquet, Véronique Legault, Alan A. Cohen, and Tony D. Williams

Table of contents

[Abbreviations 3](#_Toc515530416)

[1. Physiological assay methods and quality control 3](#_Toc515530417)

[1.1 Aerobic/metabolic capacity 3](#_Toc515530418)

[1.2 Oxidative stress/muscle damage 4](#_Toc515530419)

[1.3 Intermediary metabolism/energy supply 4](#_Toc515530420)

[1.4 Immune function 5](#_Toc515530421)

[2. Correlation between sampling time and physiological variables 5](#_Toc515530422)

[3. Pre-treatment of physiological variables 5](#_Toc515530423)

[3.1 Transformation and standardisation of physiological variables 5](#_Toc515530424)

[3.2 PCA on AG and lysis, and OXY and dROMs variables 6](#_Toc515530425)

[4. DM calculation 6](#_Toc515530426)

[4.1 Effect of sample size and centroid used in DM calculation 6](#_Toc515530427)

[4.2 Creation of multiple versions of DM – centroid definition 6](#_Toc515530428)

[4.2.1 *A priori* knowledge to identify the centroid 7](#_Toc515530429)

[4.2.2 Empirical validation of the optimum centroid 7](#_Toc515530430)

[5. PCA on physiological variables 8](#_Toc515530431)

[5.1 PCA with all physiological variables 8](#_Toc515530432)

[5.2 PCA among functional groups 8](#_Toc515530433)

[6. Pre-treatment of performance variables 8](#_Toc515530434)

[6.1 Removal of « 0 » values in reproduction variables 8](#_Toc515530435)

[6.2 PCA on performance variables 8](#_Toc515530436)

[7. Effects of physiology on performance 9](#_Toc515530437)

[7.1 Regression models 9](#_Toc515530438)

[7.2 Graphical representation 10](#_Toc515530439)

[References 10](#_Toc515530440)

[Supplementary Tables 12](#_Toc515530441)

[Supplementary Figures 18](#_Toc515530442)

# Abbreviations

***AG***: Agglutination score from NAb assays.

***BSF***: Brood size at fledging.

***BSF1***: BSF for first brood only.

***BSF2***: BSF for second brood only.

***BSF sum 2yrs***: Total number of offspring that fledged in both years.

***CK***: Creatine kinase concentrations, (U/L).

***Cort***: Corticosterone (ng/mL).

***DM****:* A measure of body condition based on statistical distance (Mahalanobis distance).

***DM v0***: DM calculated using the mean as the centroid for each biomarker.

***DM v1-v4***: DM versions based on a priori hypotheses, either using the mean + 3 standard deviations (high) or the mean – 3 standard deviations (low).

***DM v5***: DM version generated by random choice of mean, high (mean + 3 standard deviations), or low (mean - 3 standard deviations) for the centroid definition.

***dROMs***: Reactive oxygen metabolites (mg H2O2/dL).

***NAb***: Natural antibody titers

***NAb PC1***: First PCA axis on AG and lysis variables, reflecting the immune function.

***NEFA***: Non-esterified fatty acids (mmol/L).

***OXY***: Antioxidants titers (µmol HClO/mL).

***PCA***: Principal components analysis.

# Physiological assay methods and quality control

Assays for all physiological traits were conducted based on standard methods and/or previously published papers, as described below:

## Aerobic/metabolic capacity

**Hematocrit** (% packed cell volume): hematocrit was measured, following centrifugation for 3 min at 13,000 *g*, using digital calipers (± 0.01 mm) and determined as percentage of packed red cell volume to total column height (plasma plus packed red cell volume). All hematocrit measurements were made within 3 h of blood sampling, and samples were kept cool until measurement (Williams et al., 2004). Average difference between duplicate measurements: 0.1%.

**Hemoglobin** (g.dl^-1^ whole blood): was measured using the cyanomethemoglobin method (Drabkin and Austin, 1932) modified for use with a microplate spectrophotometer (BioTek Powerwave 340, BioTek Instruments, Winooski, VT, USA), using 5μl whole blood diluted in 1.25 ml Drabkin’s reagent (D5941 Sigma-Aldrich Canada, Oakville, Ontario, Canada) with absorbance measured at 540 nm.(following Wagner et al. (2008)). Mean intra-assay coefficient of variation was 0.9% and mean inter-assay coefficient of variation was 2.2% for all assays run in both years.

**Reticulocytes** (% immature red blood cells): number of immature red blood cells/total red blood cells) was estimated from whole blood smears after supravital staining with new Methylene Blue (R4132, Sigma Aldrich Canada, Oakville, Ontario, Canada). A total of 1000 red blood cells were counted per slide, and reticulocytes were distinguished from mature erythrocytes by their relatively larger size and less condensed chromatin (Campbell and Ellis, 2007) and if at least five reticulum (RNA) aggregations were visible in the cytoplasm or if a distinct ring of reticulum was surrounding the nucleus (Fernandez and Grindem, 2006). The same individual counted all blood smears (James Hou, undergraduate student). Cells were counted ten times and the average %CV was 46%.

**Corticosterone** (ng.ml^-1^; Cort): baseline Cort (see text) was measured by Dr Oliver Love, University of Windsor, using a commercially-available enzyme immune-assay (EIA; Assay Designs, Ann Arbor, MI, USA), previously validated European starlings in Love et al. (2005). All samples were run in triplicate at a 1:20 dilution with 1.5% steroid displacement buffer by volume, and samples were randomized (i.e., samples were not run in the order in which they were collected). Each plate included a control of serially diluted laying hen plasma (Sigma–Aldrich Canada, Oakville, Ontario, Canada) and a kit-provided standard curve (200,000 pg/mL). Plates were read at 405 nm using a Biotek Powerwave HT microplate reader, and the intra- and inter-assay coefficient of variation was 4.2% and 3.9% respectively.

## Oxidative stress/muscle damage

**Total antioxidant titers** (µmol HClO ml^-1^; OXY) were determined using the OXY kit from Diacron International, Italy (catalog number MC435), modified after Guindre-Parker et al. (2013). Mean intra assay coefficient of variation was 5.3% and mean interassay variation was 9.7%. Mean intra- and inter-assay coefficient of variation was 5.0% and 8.3% respectively.

**Reactive oxygen metabolites** (mg H_2_O_2_ dl^-1^; dROMs) were measured using the dROM kit from Diacron International, Italy (catalog number MC002), modified after Guindre-Parker et al. (2013). Mean intra- and inter-assay coefficient of variation was 6.2% and 11.4% respectively.

**Creatine kinase** (U.L^-1^; CK) was assayed in duplicate samples using BioAssay Systems kit ECPK-100 following the manufacturer’s guidelines with 10 µl sample volume. Mean intra- and inter-assay coefficient of variation was 4.2% and 7.5% respectively.

## Intermediary metabolism/energy supply

**Non-esterified fatty acids** (mmol.l^-1^; NEFA) were measured with the HR-2 kit (Wako Diagnostics, Mountain View, CA) following the manufacturers guidelines. Samples were assayed in duplicate using 5 µl sample volume following 1:2 (2013) or 1:3 (2014) dilution with dd H_2_O. Mean intra- and inter-assay coefficient of variation was 4.7% and 7.2% respectively.

**Triglyceride** (mmol.l^-1^) levels were measured with a colorimetric assay from Sigma-Aldrich Co. (catalog numbers F6428 and T2449), using the manufacturer’s guidelines (see Seaman et al. (2005), Williams et al. (2007)). Samples were assayed in duplicate using 5 µl sample volume following 1:2 (2013) or 1:3 (2014) dilution with dd H_2_O. Mean intra- and inter-assay coefficient of variation was 5.4% and 5.0% respectively.

**Uric acid** (mg.dL^-1^) were measured with the Quantichrom assay (DIUA-250) following the manufacturer’s guidelines. Samples were assayed in duplicate using 5 µl sample volume following 1:2 (2013) or 1:3 (2014) dilution with dd H_2_O. Mean intra- and inter-assay coefficient of variation was 2.5% and 4.4% respectively.

**Glucose** (mmol.L^-1^) levels were measured in the field with a handheld glucose meter from Accu-chek Aviva ®.

## Immune function

**Haptoglobin** (mg.ml^-1^) was assayed in duplicate using the TP801 kit from TriDelta Development, Ltd. We decreased sample volumes to 5 µl, after Zylberberg et al. (2012) and decreased the other reagents accordingly (2/3 reduction). Mean intra- and inter-assay coefficient of variation was 6.8% and 8.9% respectively for assays from both years.

**Natural antibody** (NAb) titers were assayed using the hemolysis-hemoagglutination assay developed by Matson (2005) and modified by Zylberberg (2012) for small volumes (10 µl). Lysis and agglutination scores were counted at 90 min post removal from 37°C incubation. Scores were counted twice by the same observer (MAF); if scores different more than 1.5 points, they were counted a third time and the mean of scores are reported e.g. (Matson et al., 2012).

# Correlation between sampling time and physiological variables

During the incubation period, sampling was conducted by plugging the hole of the next box early in the morning before adults left to forage and then returning to sample individuals once all nests were plugged. Accordingly, time until sampling varied across individuals, and it was possible that individuals with longer wait times might become stressed and show different physiological profiles. We looked at the correlation between the time (in minutes) from when box was plugged early in the morning to when bird was removed for sampling/measuring, (for incubation measure only) and physiological variables without outliers (n=76). Cort was not associated with time to sampling (Figure S1). We found possible evidence of a stress response for only two physiological variables, agglutination (AG) and triglycerides (Figure S2). In both cases, several individuals sampled soon after plugging had high values never observed in individuals sampled later, although it was not the same individuals who had high levels for both variables, and these variables were themselves uncorrelated (r=-0.07, p=0.60). No correlation was found between AG or triglycerides and Cort (Figure S2). We examined plots of all physiological variables against sampling time to try to detect more subtle effects, but did not find anything. Given multiple testing issues, we felt that risk of a sampling time effect was weak at best, and did not perform any subsequent adjustments.

# Pre-treatment of physiological variables

## Transformation and standardisation of physiological variables

Log-transformations were performed on glucose, dROMs, triglycerides, uric acid, and Cort. Square-root-transformations were performed on CK, haptoglobin, NEFA, and reticulocytes. All variables were then standardized (subtract mean, divide by standard deviation) to ensure equal weighting in subsequent analyses. The only variable presenting concerns about outliers after transformation was Cort, with seven raw values higher than 80 ng/ml. We conducted many of our analyses with and without these outliers but found little substantive difference, and we thus present analyses with these outliers included, with the exception that we excluded them from calculation of the mean and the variance covariance matrix used in DM calculation. This ensures that Cort “norms” are based on non-stressed individuals. Cort outliers did not have unusual levels of any other biomarkers (data not shown).

The four groups in our data (2013 incubation, 2014 incubation, 2013 chick-rearing, and 2014 chick-rearing) have sample sizes of 41, 55, 43, and 31, respectively. These sample sizes are too small to reliably use the mean of each group to center data, so we centered each physiological variable by group (year-stage) using the mean obtained with the following regression (with lmer function): biomarker ~ stage + year + (1 |Band). This provides us a larger sample size to calculate the mean while controlling for multiple samples of a given individual. Then, we divided each variable by its standard deviation (SD).

## PCA on AG and lysis, and OXY and dROMs variables

In order to understand the variation in oxidative stress and immune function and potentially reduce the number of variables, we performed separate 2-variable PCA analysis on OXY and dROMs measures (oxidative stress) and the AG and lysis variables (immune function). In both cases, we detected a similar and surprising structure: the first axis loaded in the same direction with both variables (78% and 62% of variance explained, respectively; see Figure S3), whereas the second axis explained the contrast between the variables (one negative and one positive loading). This was particularly surprising for OXY-dROMs, where we expected the first axis to explain the contrast, with high OXY and low dROMs indicating “good” oxidative status; instead, our axes appear to indicate that OXY tracks dROMs, with high on both presumably indicating a lack of control of dROMs and concomitant up-regulation of OXY (Figure S3, panel b). For both the immune and oxidative stress variables, we used the two PCA axes rather than the original variables because the biological interpretation is clearer: PCA1 is activation of the system (higher levels of both markers) and PCA2 is balance of the system (relative levels). In both cases, we also tested ratios of the raw variables and found they were strongly associated with PCA2, confirming this interpretation (Figure S3).

# DM calculation

## Effect of sample size and centroid used in DM calculation

To assess the effect of sample size on DM calculation, we used four different sample sizes to calculate the mean and variance-covariance matrix (100, 50, 30, and 20), each randomly chosen and repeated 10 times (Figure S4). We found that DM calculated with sample sizes much below 100 becomes unstable and subject to substantial sampling effects. We evaluated the effect of using all data or sub-groups of data to calculate the mean and variance-covariance matrix (Sx) used in the DM formula (Figure S5).

## Creation of multiple versions of DM – centroid definition

As noted in the main text, identifying an appropriate centroid for DM is more difficult in this context than in previous applications of our approach because many of the biomarkers here are suspected to have optimal values closer to the maximum or minimum rather than the mean. We thus defined multiple versions of DM using different criteria to identify the centroid. The simplest version, v0, uses the mean of all variables. Several alternative methods for choosing which variables should use a mean and which should use a maximum or minimum are described below. A weakness of this approach is that there is no way to know which model is the “correct” one, and it would be hoped that this might be inferred from clear results for one model and no signal for the others.

Because all variables are transformed to have a standard normal distribution, there are challenges in identifying the centroid when higher or lower values are considered optimal. Because DM is a distance from a multivariate centroid, using -∞ or ∞ as an optimum is not possible because it would give infinite weight to variables with non-mean centroids. However, using the observed maximum or minimum value is also not ideal because sampling error plays a large role in how far this value is from the mean, and thus the effective variable weight is too sensitive to sampling effects. We thus tested alternative versions that used the observed max/min, or used ±2 or ±3 standard deviations from the mean (as appropriate for max or min). We finally settled on ±3 standard deviations from the mean to identify the centroid for variables where high or low values were considered optimal. This was applied in all cases below.

### *A priori* knowledge to identify the centroid

We started by using the mean for each variable (v0) and then changed some centroids based on *a priori* hypotheses about the “optimum” values for each variable (v1-v5, Table S2). V1 supposes that the mean is optimum unless we have a very strong hypothesis to the contrary, such as that low oxidative stress is good. V2-v4 use our best guess for each variable, but incorporate different optima for cort and uric acid, where we believed there were reasonable hypotheses in both directions. V2 uses the mean for both cort and uric acid, and v3 and v4 each use one with the minimum and the other with the maximum. V5 was generated by simulation, choosing randomly mean, minimal or maximal values as the optimum (see below). (Alternative versions not shown here used other combinations for cort and uric acid; overall results did not differ in any important sense.)

### Empirical validation of the optimum centroid

One approach to identifying the appropriate centroid is to search randomly across all possible combinations of high, mean, or low optima for all variables, and identify for each variable which choice gives the strongest signal for predicting the performance variables we have. To do this, we first randomly chose the minimum, mean or maximum as the “optimum” for each physiological variable. Then, we calculated the DM with this centroid for each complete observation, and then calculated the correlation between DM and nine different performance variables. We repeated this 50,000 times. Afterwards, correlation coefficients were used to calculate z values with the following equation: z = 0.5 * log((1+r) / (1-r)). (This transformation makes the correlation coefficients approximately normally distributed, thus facilitating use in a subsequent regression.) Then, we performed a linear regression (lm function) on the z value as a function of the biomarker, to see which option among minimum, mean and maximum yields the strongest correlations. Table S3 shows which centroid measure is optimal for each combination of biomarker and performance variable. We decided to keep the optimal centroid option when the same choice was optimal for at least seven of the nine performance variables, and used the mean for all other biomarkers. This process was used to obtain v5. As can be seen in Table S3, results of this analysis were far from conclusive, with surprisingly few mean values giving a strong signal, but with the maximum or minimum varying markedly depending on the performance variable.

# PCA on physiological variables

## PCA with all physiological variables

The first axes of PCA with all physiological traits (directional and deviational, see Figure S7) explained only 14% and 13% of the variance respectively, and 6/14 of the axes had eigenvalues greater than 1 in both cases, suggesting minimal efficacy of the PCA at reducing the dimensionality. In addition to the directional and deviational PCAs, we performed PCA using all variables used in these two models (28 variables in total), but results were not much more informative (data not shown). The large number of variables relative to the sample size made the axis structure unstable, and we did not pursue these analyses further.

## PCA among functional groups

We also regrouped physiological variables into five functional groups and performed separate PCA with each group (see legend of Figure S8). However, only the first axis of three PCAs were retained in subsequent analyses: 1) the first axis of intermediary metabolism and energy supply (“metabolism/energy”) explained 37% of the variance and contraposed glucose (+) against the other three (-); 2) the first axis of aerobic/metabolic capacity (“aerobic capacity”) explained 36% of the variance and contraposed Cort against the other three; and 3) the first axis of oxidative stress and muscle damage (“oxidative stress”) explained 63% of the variance and strongly contraposed CK against OXY and dROMs. For the fourth and fifth groups (Figure S8, panels d-e), we initially included the two first axes of PCAs with OXY and dROMs, and AG and lysis, respectively, but since these axes are independent by definition, we chose to use individual variables instead.

# Pre-treatment of performance variables

## Removal of « 0 » values in reproduction variables

Reproduction variables often have many zero values and we don’t always know if this failure is due to low individual quality or to random events such as nest predation. As a sensitivity analysis we created new variables treating 0s as missing values to see if it would make a difference. This rarely had an important effect on our results and did not change our overall conclusions, so we do not present these versions.

## PCA on performance variables

Many of our variables on current breeding productivity were redundant. We thus used PCA to attempt to extract a stronger signal, we had to exclude second brood success and attempt at second brood from the PCA because they had respectively too many missing values and too little variation (51 out of 56 observations were equal to yes). The following performance variables were included in the PCA: mean fledge mass, brood size at day 6, BSF, and total offspring in current year (Figure S9). The first PCA axis on current breeding productivity explains 58% of the variance and is strongly negatively associated with the 3 variables describing number of young, but not fledgling mass. The second axis explains 26% of the variance and is nearly entirely explained by mass, which loads at 0.978.

# Effects of physiology on performance

To illustrate associations between DM and performance measures, we used correlation matrices showing correlation coefficients and p-values, using four different data subsets: all observations, observations at incubation stage only, observations at chick-rearing stage only (first and second brood), and observations at the last measure available for chick-rearing stage (first or second brood). Figure S10 shows correlations between DM calculated from all available observations and all performance variables, as an example. Note that some variables are not continuous, but the figure nonetheless serves as a useful visual guide.

## Regression models

We performed mixed-effects regressions between each performance variable and each DM version, controlling for year as a fixed effect and individual as a random effect. Performance measures with normal distributions were standardized. To facilitate comparison across results, we performed linear regressions with performance variables that had a Poisson distribution, since we found it hard to compare otherwise. Models did not converge for binomial variables (data not shown). Results of Poisson and linear regression were qualitatively similar for count variables such as number of young fledged. The function we used (lmer) does not calculate p-values; we approximated p-values by averaging the p-values calculated by taking the degrees of freedom based on the number of individuals and the degrees of freedom based on the number of data points; in this context, the difference is minimal and precise p-values are not important.

Temporal scale of variables was a challenge in regression models. For example, an individual might have DM measured three times in a year (incubation, first brood chick-rearing, second brood chick-rearing) but would only have one value for number of young fledged in the first brood or total young fledged for the year. We took three different approaches to this problem. First, we considered values for the incubation stage only, to see if DM measures at this stage might be associated with any performance variable (Fig. 4). Second, we considered values for the chick-rearing stage only (Fig. 4). However, there were three possibilities for chick-rearing measures, depending on each performance variable: 1) variables with values for first but not second brood, in that case we used the first brood measures; 2) variables with different values for first and second broods, in which case we included both values; and 3) variables with identical values for first and second broods, in which case we only considered values from the last brood (see Table S6). Third, we calculated the mean for all available DM values for one individual per year, which provide a general estimation of DM over one year. We then compared this “mean” value with related measures at the incubation stage, with a weight corresponding to the square-root of the number of DM measures used in mean calculation (Fig. 4). We chose to include values for one stage only to avoid repeated measures, and chose the incubation stage because it has the largest number of values.

We used different linear models depending on the number of complete observations for each variable and the number of individuals: 1) we did not perform any analysis for variables with less than five complete observations; 2) we used multilevel linear regressions (lmer function from lme4 package) for variables with at least 20 complete observations AND a difference greater than four between the number or individuals and observations, controlling for year as a fixed effect and individual as a random effect; and 3) we used linear regressions (lm function) for variables with less than 20 complete observations OR a difference inferior to four between the number or individuals and observations, controlling for year. The lmer function does not directly provide p-values. We thus used the t value to calculate the maximum and minimum p-values, with the following equation: 2 * (1 - pt(abs(t.value),df). For the maximum, we used the number of observations-1 as df and for the minimum, the number of individuals-1. We took the mean of these two values. The differences between the maximum and minimum p-values are always relatively small, which was verified for the seven lowest p-values. For example, the lowest p-value has a minimum and a maximum of 0.00010848 and 0.00013936 respectively.

## Graphical representation

We present the result for each regression analysis with a box. The height of the horizontal line in the box represents the effect size, with the following 10 breakpoints: 0, 0.01, 0.05, 0.1, 0.2, 0.4, 0.7, 1, 1.5, 2 and 5. The color underneath is blue for positive effects and red for negative ones, and the hue represents p-values, according to the following breakpoints from dark to light color: 0, 1e-08, 1e-05, 0.01, 0.05, 0.1, 1.

# References

Campbell, T.W., Ellis, C., 2007. Avian and Exotic Animal Hematology and Cytology, 3rd edn. ed. Iowa State Press, Ames, Iowa.

Drabkin, D.L., Austin, J.H., 1932. Spectrophotometric studies: spectrophotometric constants for common hemoglobin derivatives in human, dog and rabbit blood. Journal of Biological Chemistry 98, 719-733.

Fernandez, F.R., Grindem, C.B., 2006. Reticulocyte response, in: B.F. Feldman, J.G. Zinkl, N.C. Jain (Eds.), Schalmʼs Veterinary Hematology, 5th edn. ed. Blackwell, Ames, Iowa, 110-116.

Guindre-Parker, S., Baldo, S., Gilchrist, H.G., Macdonald, C.A., Harris, C.M., Love, O.P., 2013. The oxidative costs of territory quality and offspring provisioning. Journal of Evolutionary Biology 26, 2558-2565.

Love, O.P., Chin, E.H., Wynne-Edwards, K.E., Williams, T.D., 2005. Stress Hormones: A Link between Maternal Condition and Sex-Biased Reproductive Investment. American Naturalist 166, 751-766.

Matson, K.D., Horrocks, N.P.C., Versteegh, M.A., Tieleman, B.I., 2012. Baseline haptoglobin concentrations are repeatable and predictive of certain aspects of a subsequent experimentally-induced inflammatory response. Comparative Biochemistry and Physiology Part A: Molecular & Integrative Physiology 162, 7-15.

Matson, K.D., Ricklefs, R.E., Klasing, K.C., 2005. A hemolysis–hemagglutination assay for characterizing constitutive innate humoral immunity in wild and domestic birds. Developmental & Comparative Immunology 29, 275-286.

Seaman, D.A., Guglielmo, C.G., Williams, T.D., 2005. Effects of physiological state, mass change and diet on plasma metabolite profiles in the western sandpiper Calidris mauri. Journal of Experimental Biology 208, 761-769.

Wagner, E.C., Stables, C.A., Williams, T.D., 2008. Hematological changes associated with egg production: direct evidence for changes in erythropoiesis but a lack of resource-dependence. Journal of Experimental Biology 211, 2960-2968.

Williams, T.D., Challenger, W.O., Christians, J.K., Evanson, M., Love, O., Vezina, F., 2004. What causes the decrease in haematocrit during egg production? Functional Ecology 18, 330-336.

Williams, T.D., Warnock, N., Takekawa, J., Bishop, M.A., 2007. Flyway scale variation in plasma triglyceride levels as an index of refueling rate in spring migrating Western Sandpipers. Auk 124, 886-897.

Zylberberg, M., Lee, K.A., Klasing, K.C., Wikelski, M., 2012. Increasing avian pox prevalence varies by species, and with immune function, in Galápagos finches. Biological Conservation 153, 72-79.

# Supplementary Tables

**Table S1.** Raw data values for body mass and physiological traits for female European starlings sampled during incubation and during chick-rearing for first and second broods in 2013 and 2014. Values are means ± S.D.

| **Trait** | **Year = 2013** | | | **Year = 2014** | | |
| --- | --- | --- | --- | --- | --- | --- |
|  | **Incubation** | **1^st^ brood** | **2^nd^ brood** | **Incubation** | **1^st^ brood** | **2^nd^ brood** |
| **Mass (g)** | 80.8 ± 3.5  (46) | 80.5 ± 3.3  (30) | 79.3 ± 4.2  (12) | 81.9 ± 3.6  (30) | 80.7 ± 3.9  (24) | 79.7 ± 2.5  (7) |
| **Hct (%, PCV)** | 52.9 ± 3.0  (46) | 51.5 ± 3.6  (31) | 50.3 ± 2.9  (14) | 52.9 ± 3.3  (30) | 52.5 ± 2.2  (24) | 50.4 ± 3.7  (7) |
| **Hb (g.dl^-1^)** | 16.2 ± 2.5  (46) | 16.6 ± 2.0  (30) | 15.6 ± 1.0  (14) | 14.2 ± 2.4  (30) | 15.5 ± 1.8  (24) | 14.9 ± 1.4  (7) |
| **Reticulocyte (%)** | 15.6 ± 6.1  (44) | 8.2 ±5.1  (28) | 5.4 ± 4.8  (5) | 8.7 ± 5.9  (26) | 7.3 ± 2.5  (11) | 2.9 ± 1.8  (5) |
| **Cort (ng.ml^-1^)** | 24.1 ± 14.0  (27) | 20.9 ± 12.2  (23) | 18.0 ± 13.8  (9) | 27.3 ± 16.9  (30) | 23.5 ± 17.0  (23) | 16.2 ± 10.0  (7) |
| **dROMS (mg H_2_O_2_.dl^-1^)** | 2.74 ± 0.99  (43) | 3.29 ± 1.25  (30) | 2.50 ± 0.95  (13) | 1.32 ± 0.48  (30) | 1.44 ± 0.40  (24) | 1.52 ± 0.32  (7) |
| **OXY (µmol HClO.ml^-1^)** | 254.4 ± 38.4  (43) | 271.1 ± 36.8  (31) | 265.0 ± 32.1  (13) | 167.6 ± 38.2  (30) | 232.9 ± 46.5  (24) | 224.8 ± 34.8  (7) |
| **CK (U.l^-1^)** | 100.7 ± 52.6  (35) | 80.2 ± 52.1  (27) | 100.7 ± 60.5  (13) | 123.4 ± 36.2  (28) | 95.0 ± 32.4  (20) | 92.2 ± 23.2  (7) |
| **NEFA (mmol.l^-1^)** | 0.599 ± 0.397  (43) | 0.872 ± 0.413  (31) | 0.708 ± 0.367  (13) | 0.708 ± 0.309  (30) | 0.986 ± 0.360  (24) | 1.213 ± 0.729  (7) |
| **Trig (mmol.l^-1^)** | 1.16 ± 0.60  (42) | 2.14 ± 0.62  (31) | 2.00 ± 0.55  (13) | 1.089 ± 0.558  (30) | 2.17 ± 0.50  (24) | 3.12 ± 1.52  (7) |
| **Uric acid (mg.dl^-1^)** | 11.9 ± 4.67  (41) | 26.3 ± 6.9  (31) | 17.4 ± 4.4  (13) | 13.5 ± 3.5  (30) | 29.0 ±5.6  (24) | 35.2 ± 4.9  (7) |
| **Glucose (mmol.l^-1^)** | 12.8 ± 2.0  (42) | 12.4 ± 1.14  (30) | 15.0 ± 2.0  (14) | 11.7 ± 1.2  (30) | 13.1 ± 1.55  (24) | 12.9 ± 0.6  (7) |
| **Hap (mg.ml^-1^)** | 0.433 ± 0.303  (40 | 0.425 ± 0.287  (30) | 0.355 ± 0.234  (12) | 1.015 ± 0.049  (30) | 1.171 ± 0.396  (22) | 1.010 ± 0.294  (7) |
| **AG score** | 7.24 ± 0.87  (36) | 7.62 ± 1.19  (29) | 6.97 ± 0.81  (13) | 7.58 ± 1.36  (28) | 7.30 ± 1.32  (20) | 7.75 ± 1.35  (7) |
| **Lysis score** | 4.51 ± 0.98  (36) | 4.79 ± 0.79  (29) | 4.81 ± 0.89  (13) | 5.08 ± 0.57  (28) | 4.90 ± 0.70  (20) | 5.46 ± 0.78  (7) |

**Table S2.** Summaries of how various DM versions were calculated.

|  | **V0** | **V1** | **V2** | **V3** | **V4** | **V5** |
| --- | --- | --- | --- | --- | --- | --- |
| **Hematocrit** | Mean | Mean | Mean | Mean | Mean | Mean |
| **Hemoglobin** | Mean | Mean | High | High | High | High |
| **Glucose** | Mean | Mean | High | High | High | Mean |
| **NAb PC1** | Mean | Mean | Mean | Mean | Mean | Mean |
| **NAb PC2** | Mean | Mean | Mean | Mean | Mean | Mean |
| **CK** | Mean | Low | Low | Low | Low | Mean |
| **Haptoglobin** | Mean | Mean | Mean | Mean | Mean | Mean |
| **OXY-dROMs-PC1** | Mean | Low | Low | Low | Low | Mean |
| **OXY-dROMs-PC2** | Mean | Low | Low | Low | Low | Low |
| **NEFA** | Mean | High | High | High | High | Mean |
| **Triglycerides** | Mean | Mean | High | High | High | High |
| **Uric acid** | Mean | Mean | Mean | Low | High | Mean |
| **Reticulocytes (%)** | Mean | Mean | Mean | Mean | Mean | Mean |
| **Corticosterone** | Mean | Mean | Mean | High | Low | Mean |

The first version (V0) is calculated using the mean as the centroid for each biomarker. V1-V4 were generated based on *a priori* hypotheses, either using the mean + 3 standard deviations (SD; high) or the mean – 3 SDs (low). V5 was generated by random choice of min, mean or max.

**Table S3.** Optimal centroid for each physiological variable, based on their correlation with four performance measures among 50,000 random iterations.

|  | **Reproduction-**  **PC1** | **BSF**  **(no zero)** | **Mean fledge mass** | **BSF2.nextyr (no zero)** |
| --- | --- | --- | --- | --- |
| **Hematocrit** | min | min | max | max |
| **Hemoglobin** | max | max | min | max |
| **Glucose** | min | max | min | min |
| **NAb PC1** | min | min | min | min |
| **NAb PC2** | max | min | max | max |
| **CK** | min | max | min | min |
| **Haptoglobin** | max | min | max | min |
| **OXY-dROMs-PC1** | max | min | min | max |
| **OXY-dROMs-PC2** | max | min | min | min |
| **NEFA** | max | min | max | max |
| **Triglycerides** | mean | max | min | max |
| **Uric acid** | min | max | max | min |
| **Reticulocytes (%)** | min | min | mean | min |
| **Corticosterone** | max | min | min | min |

**Table S4**. Correlation matrix for 14 physiological traits and body mass in **incubating** European starlings. *n* ≥ 70 for all traits except log Cort (n = 57), sqrt CK (n = 63) and NAb PC1 (n = 64). Values are correlation coefficients (r); ***bold*** = *p* < 0.05 * Yellow indicates significant after FDR adjustment.

|  | **Aerobic/metabolic capacity** | | | | **Oxidative stress/ muscle damage** | | | **Intermediary metabolism/energy supply** | | | | **Immune function** | |  |
| --- | --- | --- | --- | --- | --- | --- | --- | --- | --- | --- | --- | --- | --- | --- |
|  | **Hct** | **Hb** | **sqrt.**  **Ret** | **log Cort** | **Oxy** | **log dROM** | **sqrt.**  **CK** | **sqrt.**  **NEFA** | **log Trig** | **log**  **Uric** | **log**  **Gluc** | **sqrt.**  **Hap** | **NAb**  **PC1** | **Body mass** |
| **Hct** | 1.00 | ***0.29*** | -0.22 | -0.21 | 0.07 | 0.06 | -0.14 | 0.03 | ***-0.27*** | -0.02 | -0.12 | -0.05 | -0.23 | 0.17 |
| **Hb** |  | 1.00 | 0.21 | -0.19 | ***0.29*** | 0.08 | -0.02 | -0.13 | ***-0.27*** | -0.23 | 0.04 | ***-0.28*** | 0.06 | 0.04 |
| **sqrt.Ret** |  |  | 1.00 | -0.24 | ***0.29*** | 0.22 | -0.20 | -0.06 | 0.17 | -0.22 | -0.12 | ***-0.50*** | ***0.28*** | **-*0.25*** |
| **log Cort** |  |  |  | 1.00 | 0.02 | -0.02 | 0.10 | 0.17 | -0.10 | -0.01 | 0.01 | -0.07 | 0.02 | 0.04 |
| **Oxy** |  |  |  |  | 1.00 | ***0.68*** | ***-0.29*** | 0.00 | 0.10 | -0.12 | 0.19 | ***-0.43*** | 0.06 | ***-0.26*** |
| **log dROM** |  |  |  |  |  | 1.00 | ***-0.32*** | -0.18 | 0.01 | ***-0.34*** | 0.06 | ***-0.49*** | -0.04 | -0.21 |
| **sqrt.CK** |  |  |  |  |  |  | 1.00 | -0.02 | -0.07 | 0.09 | 0.04 | ***0.38*** | -0.14 | 0.03 |
| **sqrt.NEFA** |  |  |  |  |  |  |  | 1.00 | ***0.31*** | ***0.31*** | 0.15 | ***0.30*** | -0.04 | -0.05 |
| **log Trig** |  |  |  |  |  |  |  |  | 1.00 | ***0.27*** | 0.15 | 0.12 | -0.01 | 0.08 |
| **log Uric** |  |  |  |  |  |  |  |  |  | 1.00 | ***0.30*** | ***0.33*** | -0.06 | 0.20 |
| **log Gluc** |  |  |  |  |  |  |  |  |  |  | 1.00 | 0.00 | -0.03 | -0.02 |
| **sqrt.Hap** |  |  |  |  |  |  |  |  |  |  |  | 1.00 | ***-0.34*** | ***0.31*** |
| **NAb PC1** |  |  |  |  |  |  |  |  |  |  |  |  | 1.00 | -0.15 |

**Table S5**. Correlation matrix for 15 physiological traits and body mass in **chick-rearing** European starlings. *n* ≥ 52 for all traits except sqrt Ret (n = 39), log Cort (n = 46) sqrt CK (n = 48) and NAb PC1 (n = 49). Values are correlation coefficients (r); **bold** = *p* < 0.05 *. Yellow indicates significant after FDR adjustment.

|  | **Aerobic/metabolic capacity** | | | | **Oxidative stress/ muscle damage** | | | **Intermediary metabolism/energy supply** | | | | **Immune function** | |  |  |
| --- | --- | --- | --- | --- | --- | --- | --- | --- | --- | --- | --- | --- | --- | --- | --- |
|  | **Hct** | **Hb** | **sqrt.**  **Ret** | **log Cort** | **Oxy** | **log dROM** | **sqrt.**  **CK** | **sqrt.**  **NEFA** | **log Trig** | **log**  **Uric** | **log**  **Gluc** | **sqrt.**  **Hap** | **NAb**  **PC1** | **Body mass** | **Wing loading** |
| **Hct** | 1.00 | ***0.42*** | -0.26 | -0.26 | 0.08 | -0.17 | 0.17 | -0.16 | 0.03 | 0.20 | 0.13 | -0.03 | ***0.32*** | -0.07 | -0.25 |
| **Hb** |  | 1.00 | 0.10 | -0.10 | ***0.30*** | 0.04 | 0.00 | -0.20 | 0.05 | 0.07 | 0.09 | -0.18 | 0.02 | -0.15 | -0.11 |
| **sqrt.Ret** |  |  | 1.00 | 0.20 | -0.10 | -0.14 | -0.13 | 0.11 | -0.07 | -0.16 | 0.09 | 0.08 | -0.18 | -0.27 | -0.25 |
| **log Cort** |  |  |  | 1.00 | -0.05 | 0.00 | 0.07 | 0.21 | -0.29 | -0.22 | 0.02 | 0.10 | -0.05 | -0.15 | 0.15 |
| **Oxy** |  |  |  |  | 1.00 | ***0.36*** | -0.05 | -0.17 | 0.10 | 0.10 | -0.17 | ***-0.33*** | -0.02 | -0.11 | 0.25 |
| **log dROM** |  |  |  |  |  | 1.00 | -0.21 | -0.11 | 0.10 | -0.25 | ***-0.29*** | ***-0.53*** | -0.28 | 0.23 | 0.25 |
| **sqrt.CK** |  |  |  |  |  |  | 1.00 | 0.11 | -0.10 | ***0.32*** | -0.15 | 0.13 | -0.08 | 0.20 | -0.01 |
| **sqrt.NEFA** |  |  |  |  |  |  |  | 1.00 | 0.13 | 0.22 | ***-0.32*** | 0.14 | 0.09 | 0.18 | 0.04 |
| **log Trig** |  |  |  |  |  |  |  |  | 1.00 | 0.18 | -0.09 | 0.15 | -0.13 | 0.10 | 0.00 |
| **log Uric** |  |  |  |  |  |  |  |  |  | 1.00 | ***-0.32*** | 0.07 | 0.14 | -0.03 | -0.05 |
| **log Gluc** |  |  |  |  |  |  |  |  |  |  | 1.00 | 0.06 | -0.03 | -0.04 | -0.15 |
| **sqrt.Hap** |  |  |  |  |  |  |  |  |  |  |  | 1.00 | -0.12 | -0.05 | -0.09 |
| **NAb PC1** |  |  |  |  |  |  |  |  |  |  |  |  | 1.00 | -0.13 | -0.14 |

**Table S6.** Availability of performance variables according to incubation and chick-rearing stages.

|  |  | **Chick-rearing** | | |  |
| --- | --- | --- | --- | --- | --- |
| **Outcome** | Incubation | First brood | Both broods | Last brood | Mean |
| **Mass** | X |  | X |  | X |
| **Tarsus** | X |  |  | X | X |
| **Wing loading** | X |  | X |  | X |
| **Brood size at day 17** | X |  | X |  | X |
| **BSF2** | X | X |  |  | X |
| **BSF sum per year** | X |  |  | X | X |
| **Mean fledge mass** | X |  | X |  | X |
| **BSF sum next year** | X |  |  | X | X |
| **BSF sum 2yrs** | X |  |  | X | X |
| **Lay date next year** | X |  |  | X | X |
| **Reproduction-PC1** | X | X |  |  | X |

# Supplementary Figures


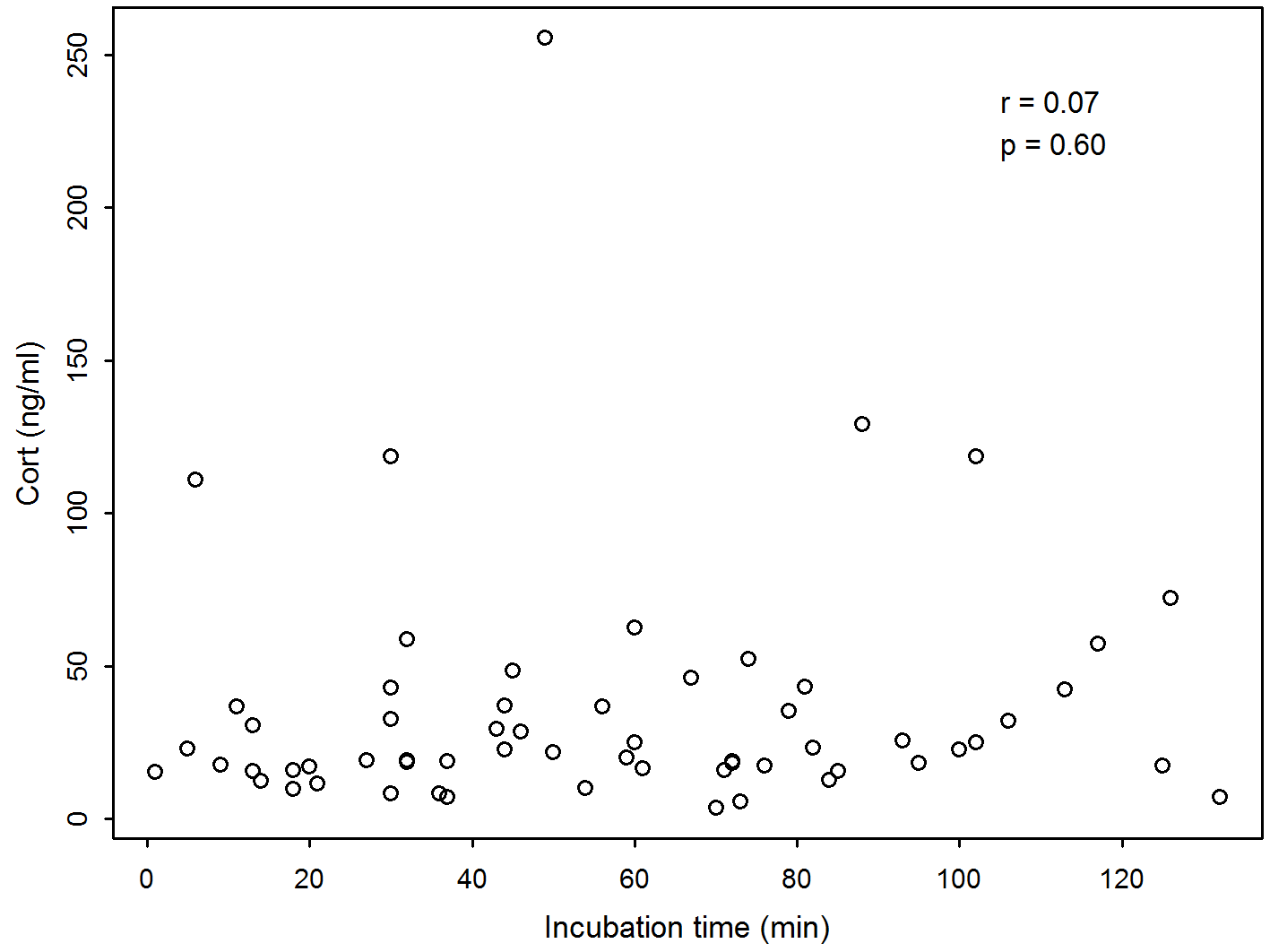


**Figure S1.** Correlations between removal time from box and corticosterone (Cort) level in incubating females (n=76).


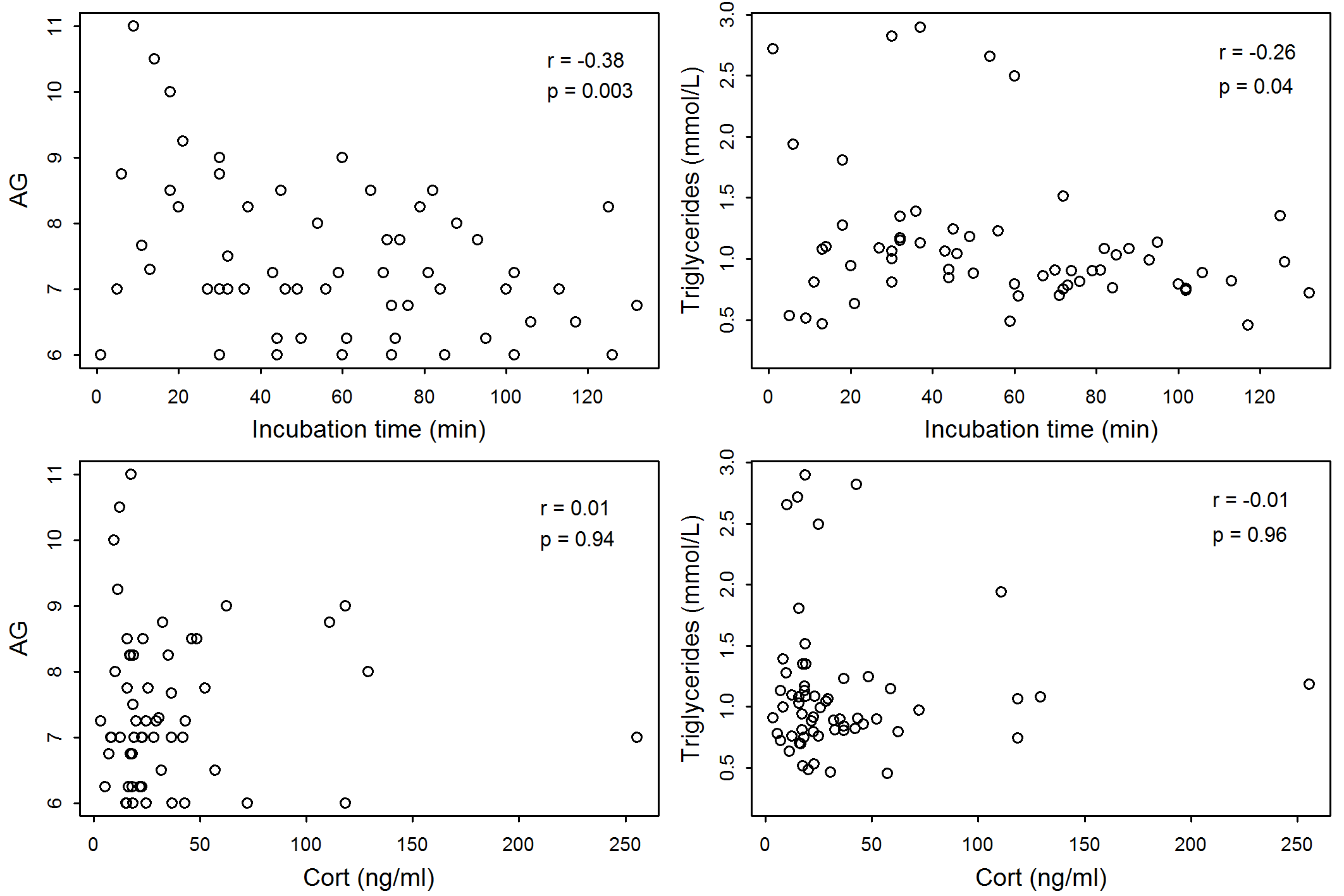


**Figure S2.** Correlations between removal time from box and AG (a) and triglycerides (b), and between Cort and AG (c) and triglycerides (d) in incubating females (n=76).

**
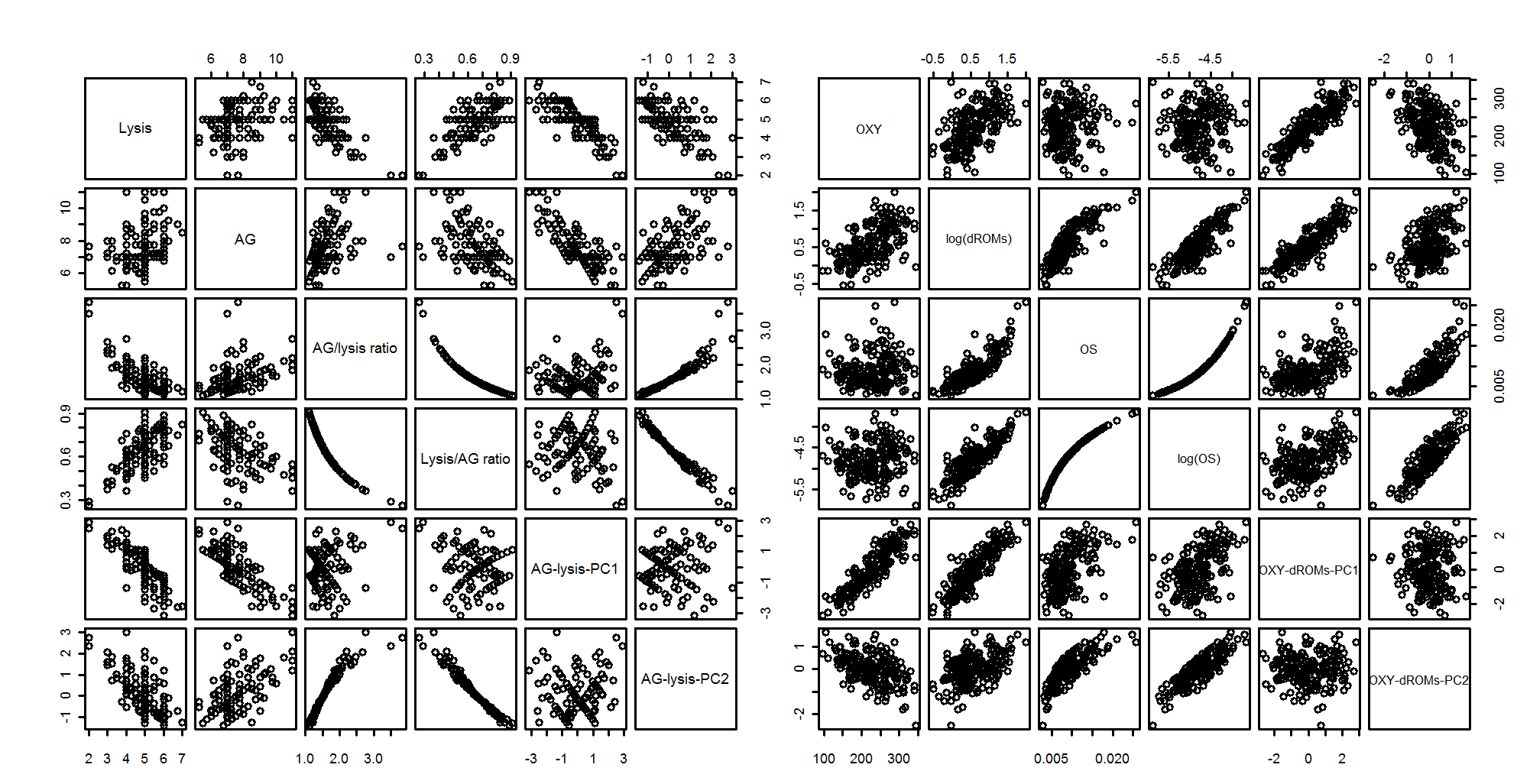
**

**Figure S3.** Correlations between raw variables and axes of a PCA. The left panel shows correlations between AG and lysis variables, their ratios and the two first axes of a PCA with them. The first PCA axis is negatively correlated with both variables, whereas the second PCA axis represents the ratio between them. The right panel shows correlations between OXY, dROMs, and oxidative stress (OS) variables (the ratio of dROMs/OXY; we also include the log of OS, i.e. log(dROMs/OXY), for comparison), and the two first axes of the PCA on OXY and dROMs. The first PCA axis is positively correlated with OXY and dROMs variables, whereas the second axis is positively correlated with OS.


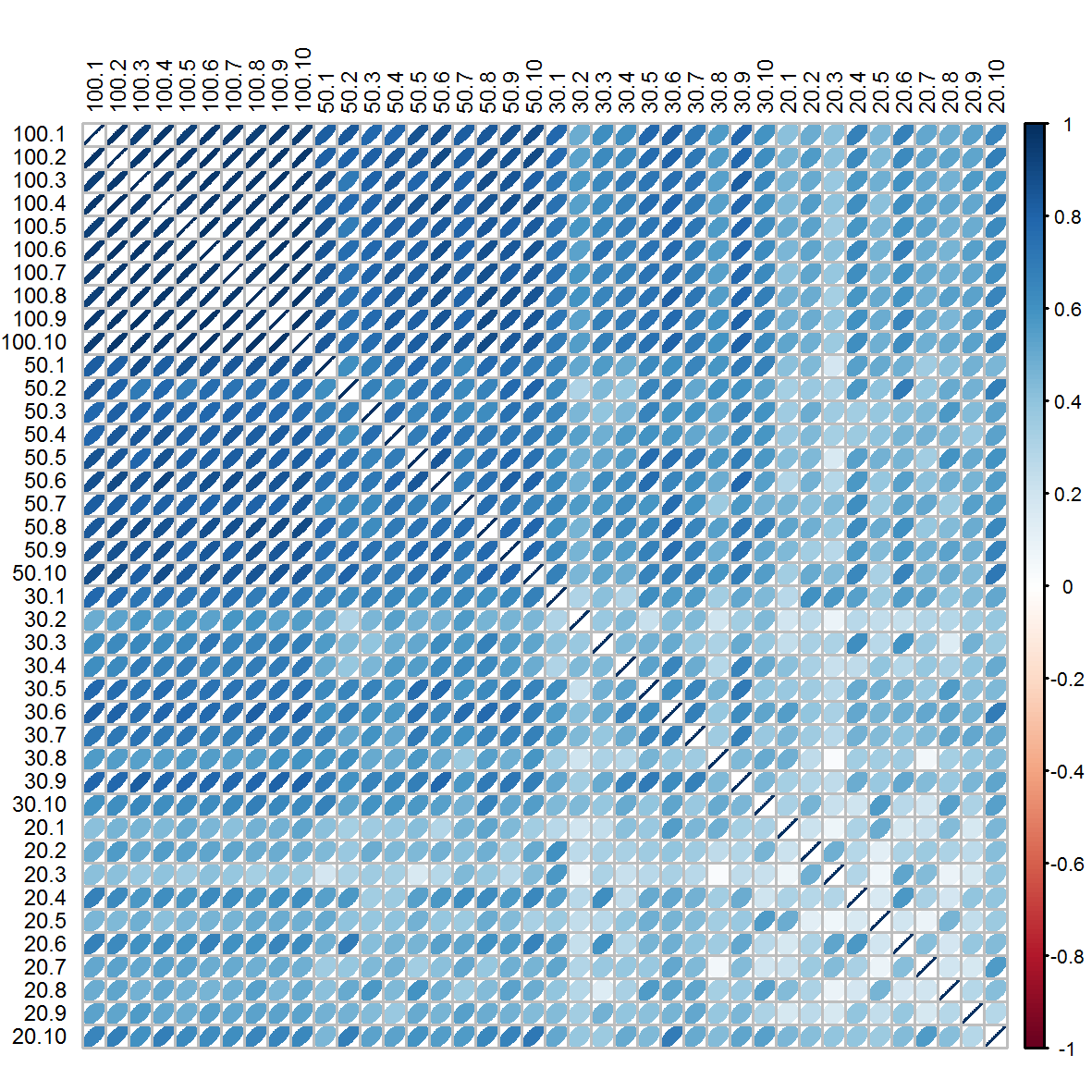


**Figure S4.** Correlations between DM calculated with different sample sizes used to estimate the mean and variance-covariance matrix (100, 50, 30, and 20). Each sample was randomly chosen and repeated 10 times. Ellipses indicate correlations visually: blue when positive, red when negative, and darker and narrower when stronger.


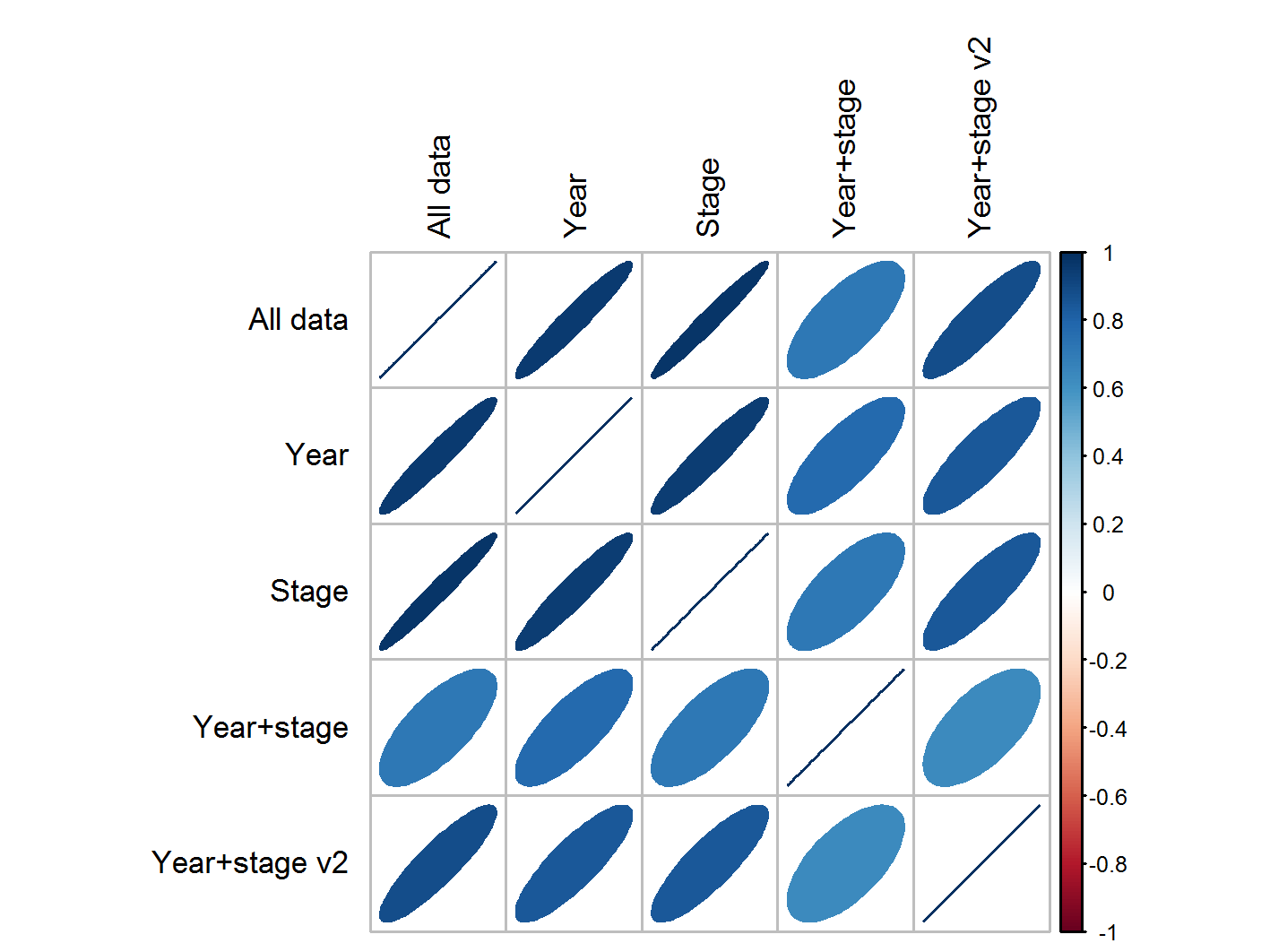


**Figure S5.** Correlations between DM values obtained with different mean and variance-covriance matrix (Sx) used in the formula. In the first column, the mean and Sx are calculated from all data. In the second column, mean and Sx were calculated separately for 2013 and 2014 groups. In the third column, mean and Sx were calculated separately for incubation and nestling stages groups. In the fourth column, we used four groups to calculate mean and Sx, based on years and stage (2013/incubation, 2014/incubation, 2013/chick-rearing, and 2014/chick-rearing). In the last column, we used the four same groups to calculate means, but we used all data to calculate Sx. Ellipses indicate correlations visually: blue when positive and darker and narrower when stronger. The poor performance (weaker correlation) when using small Sx subgroups is probably due to unstable estimation of Sx at small sample sizes.

**
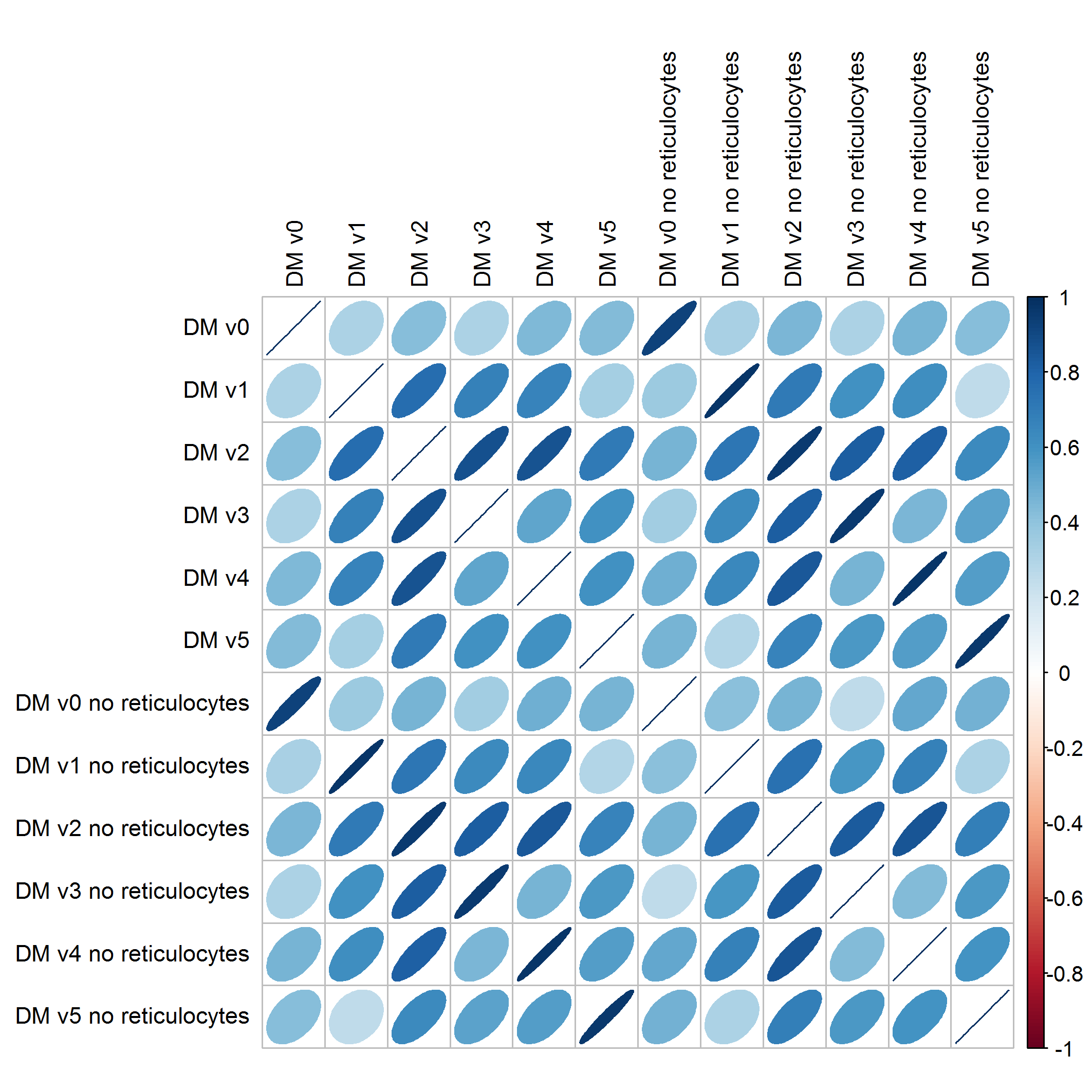
**

**Figure S6.** Correlations between the final set of DMs. The first version (V0) is calculated using the mean as the centroid for each biomarker. V1-V4 were generated based on a priori hypotheses, either using the mean + 3 standard deviations (SD; high) or the mean – 3 SDs (low). V5 was generated by random choice of min, mean or max (see text and Table S3 for details). Ellipses indicate correlations visually: blue when positive, red when negative, and darker and narrower when stronger.

**
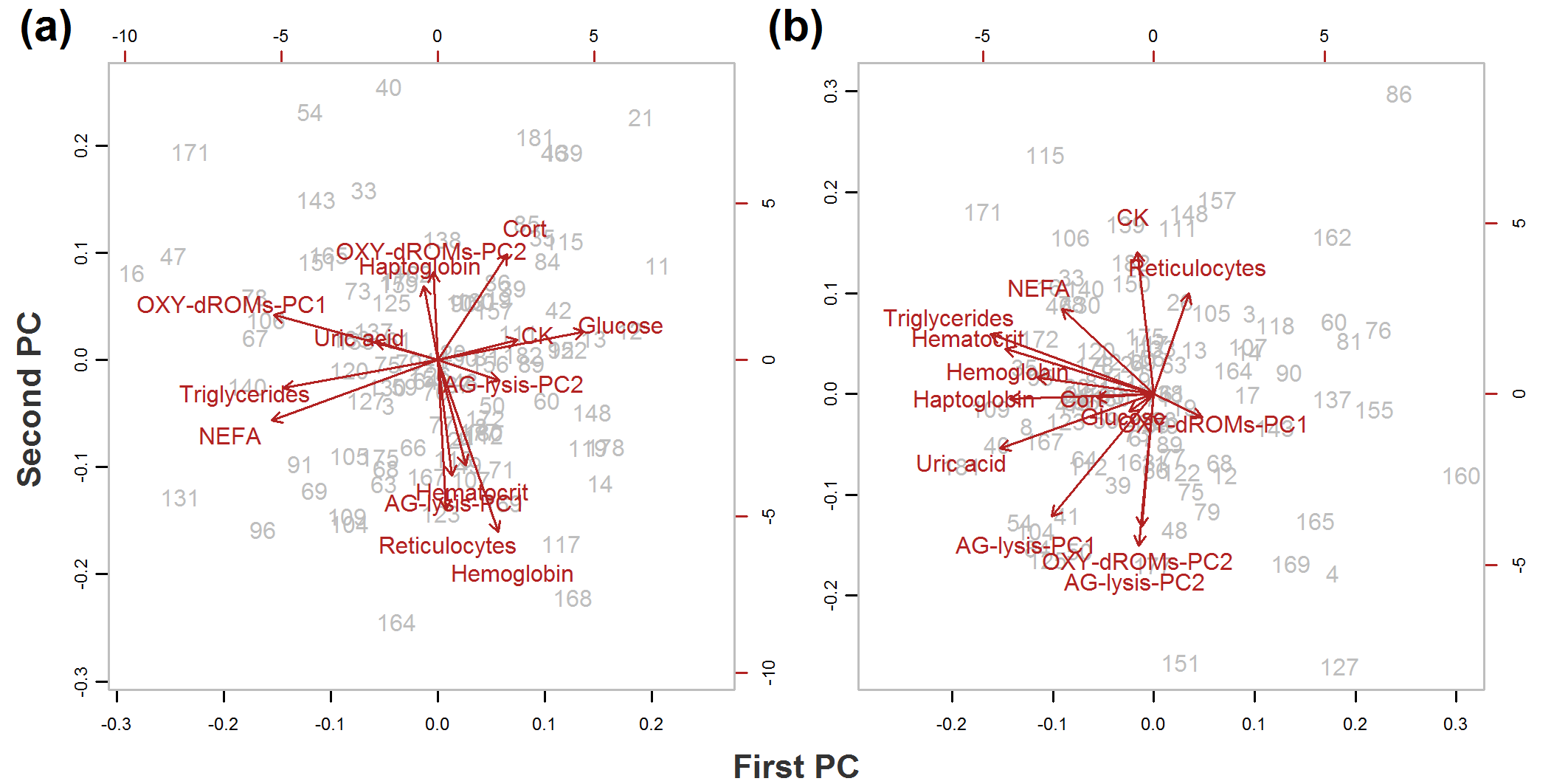
**

**Figure S7.** PCA with all physiological variables. We performed PCAs with **(a)** all available physiological variables (14 variables, n=100) centered and reduced according to year and stage, and **(b)** the logarithm of the absolute values of the former centered-reduced variables.

**
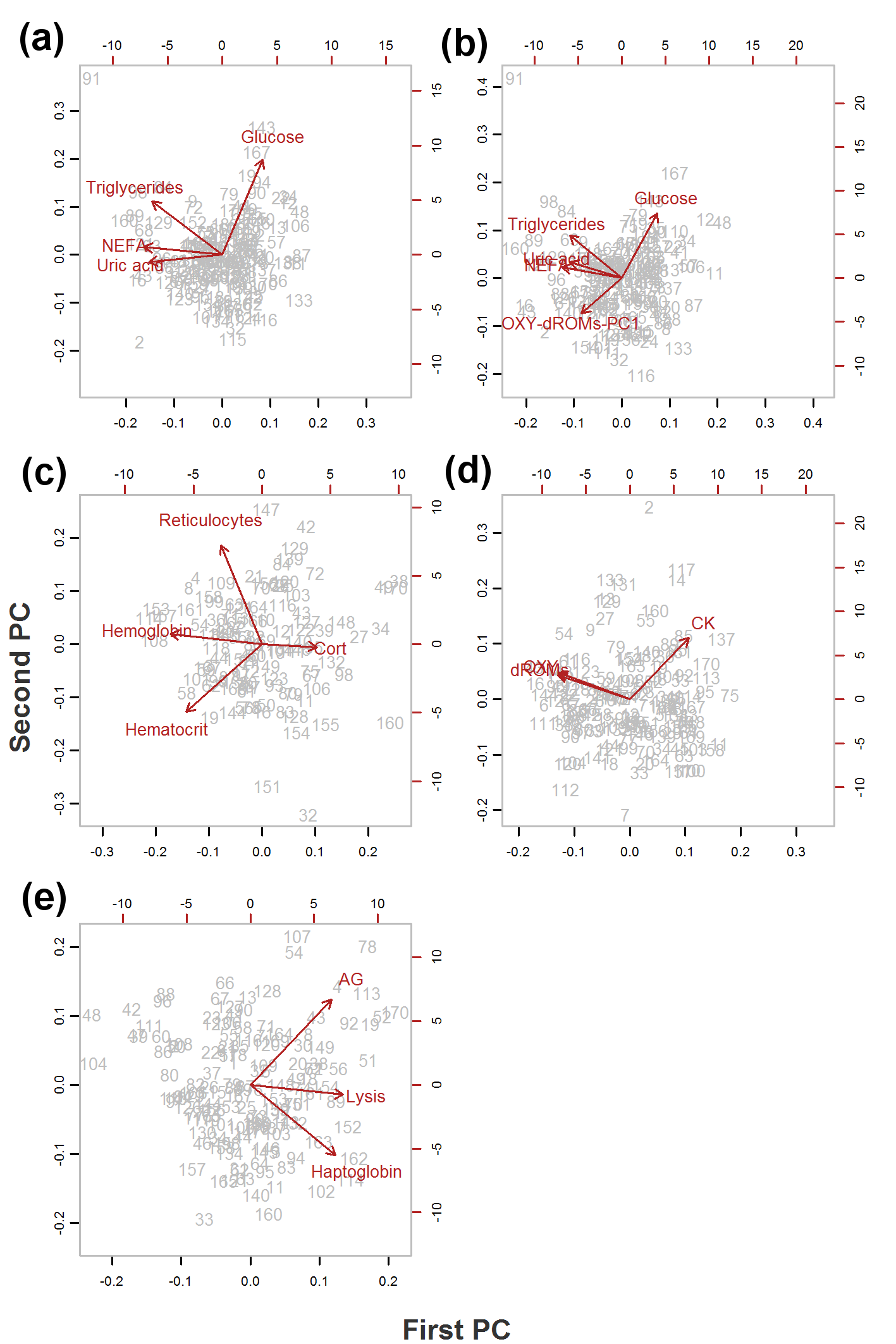
**

**Figure S8.** PCA on functional groups among physiological variables. **(a)** The first group represents intermediary metabolism and energy supply and includes the following variables: NEFA, triglycerides, uric acid, and glucose. **(b)** The second group is identical to the first, but with OXY-dROMs PC1 added. **(c)** The third group represents aerobic and metabolic capacity with the following variables included: hematocrit, hemoglobin, reticulocytes, and corticosterone. **(d)** The fourth group reflects oxidative stress and muscle damage and includes CK, OXY, and dROMs. **(e)** The last group is formed by haptoglobin and natural antibodies (AG and lysis), and represents immune function.


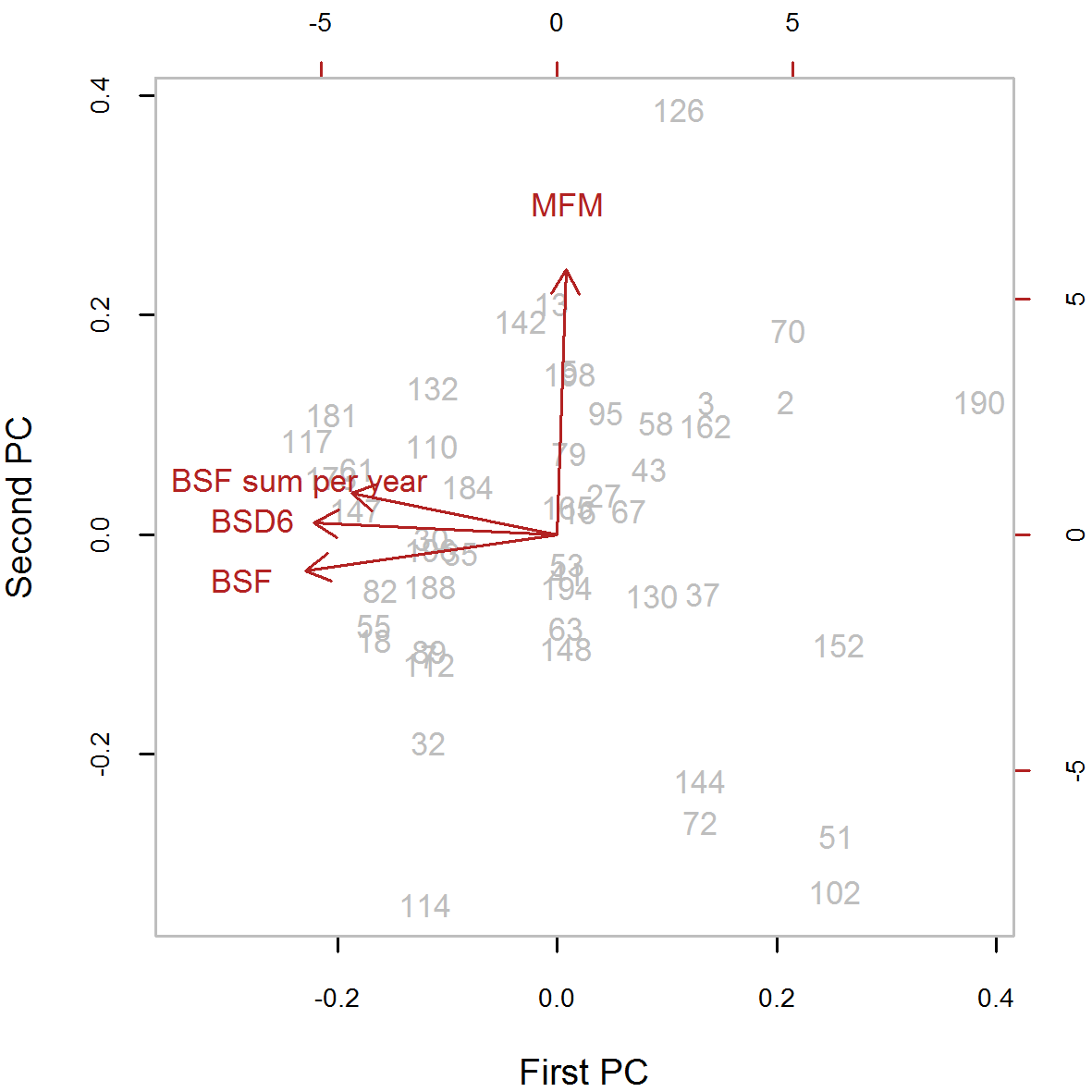


**Figure S9.** PCA on reproduction variables. The following outcome measures were included in the PCA (n=50): mean fledge mass (MFM), brood size at day 6 (BSD6), brood size at fledging (BSF), and total offspring that fledged in a year (BSF sum per year).


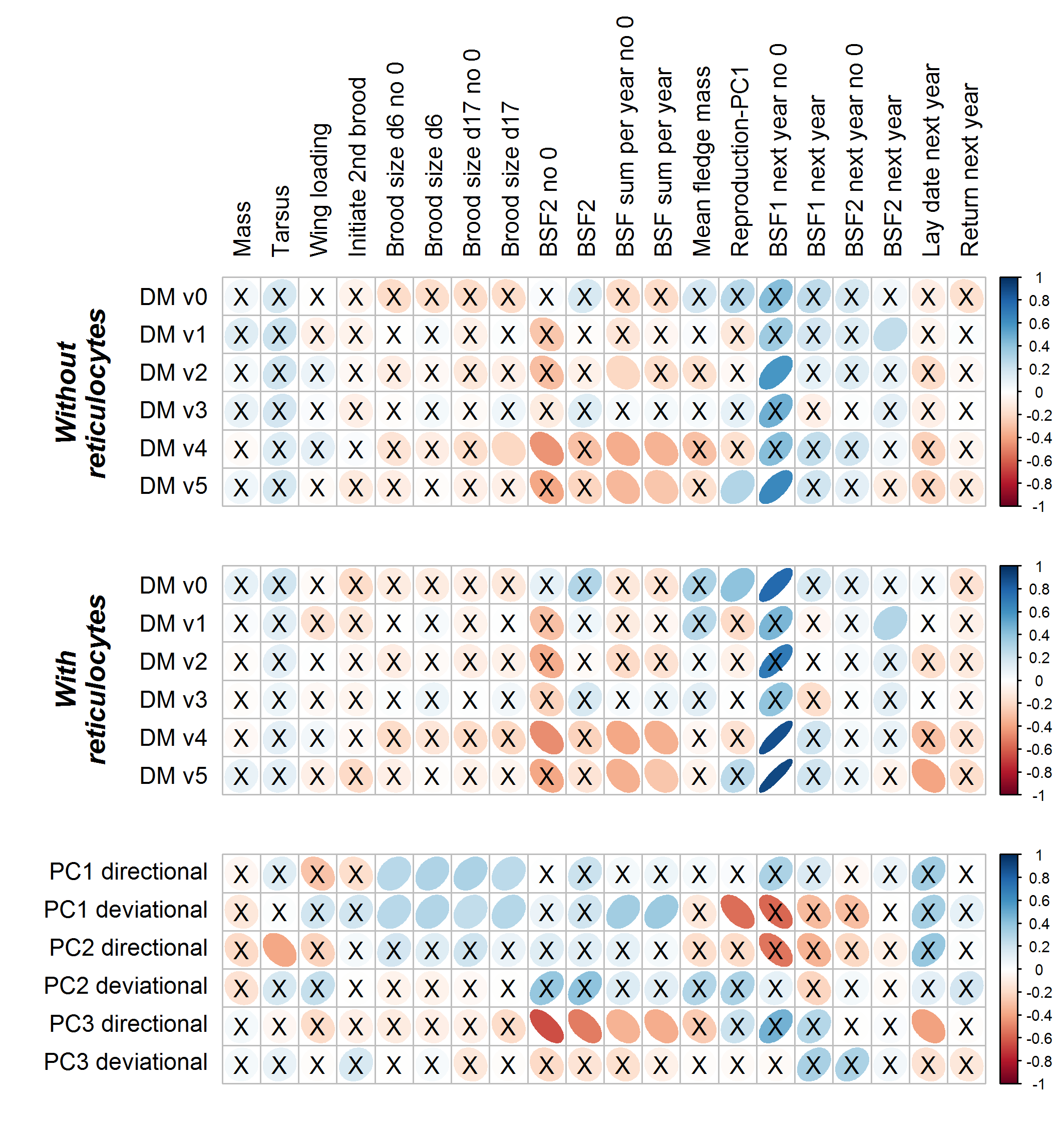


**Figure S10.** Correlations between all DM versions and all performance measures. Different versions of DM, excluding (**upper panel**) or including reticulocyte (**middle panel**). **Lower panel**: the first three axes of the PCAs on all physiological variables (directional and deviational; see Figure S7 for details). Ellipses indicate correlations visually: blue when positive, red when negative, and darker and narrower when stronger. Non-significant correlations are marked with an “X”.
